# Psychosocial determinants of handwashing and physical distancing behaviour during the COVID‐19 pandemic in the Netherlands: A longitudinal analysis

**DOI:** 10.1111/bjhp.12755

**Published:** 2024-10-02

**Authors:** Carlijn Bussemakers, Nicole Stappers, Floor Kroese, Bas van den Putte, Marijn de Bruin

**Affiliations:** ^1^ IQ Health Department Radboud University Medical Center Nijmegen The Netherlands; ^2^ Department of Social, Health and Organizational Psychology Utrecht University Utrecht The Netherlands; ^3^ Centre for Prevention, Lifestyle and Health National Institute for Public Health and the Environment (RIVM) Bilthoven The Netherlands; ^4^ Amsterdam School of Communications Research (ASCoR) University of Amsterdam Amsterdam The Netherlands

**Keywords:** COVID‐19 pandemic, dynamic cohort study, handwashing, health belief model, physical distancing, protection motivation theory, within‐person analysis

## Abstract

**Objectives:**

Physical distancing and handwashing can be important infection prevention measures during an infectious disease outbreak such as the COVID‐19 pandemic. To stimulate these behaviours, knowledge of psychosocial determinants as well as contextual factors is vital. We present longitudinal, within‐person analyses of the impact of contextual and psychosocial factors on handwashing and distancing behaviour.

**Design:**

We used individual‐level data (186,490 participants completing 971,899 surveys) from the Corona Behavioural Unit COVID‐19 Cohort, a dynamic cohort study conducted during 26 months of the COVID‐19 pandemic in the Netherlands.

**Methods:**

Fixed‐effects models were employed to estimate within‐person associations between psychosocial factors and behaviour, combined with main and moderating effects of contextual factors.

**Results:**

Pandemic severity was associated with more handwashing and distancing behaviour, while the duration of the pandemic had little effect. Within‐person changes in response efficacy were most relevant for changes in both handwashing and distancing behaviour, while self‐efficacy, descriptive norms and perceived severity of infecting others affected behaviour indirectly. These effects were stable over time. Associations were larger in cross‐sectional models, indicating that such models tend to overestimate effects.

**Conclusions:**

Our study highlights the importance of longitudinal data and within‐person models to detect possible causal associations. The results suggest that during an outbreak, government and public health professionals should clearly communicate the severity of the pandemic (e.g., hospitalization rates) and the effectiveness of recommended prevention measures in reducing that risk; and seek to improve people's capabilities and opportunities to adhere to guidelines, for example, by modifying the environment.

**Statement of contribution**

**What is already known on this topic?**

- Physical distancing and handwashing can be important infection prevention behaviours during an infectious disease outbreak such as the COVID‐19 pandemic.
- Adherence to these behavioural guidelines varied between people and over time.
- Many psychosocial factors have been associated with differences in adherence between people, rather than studying drivers of adherence within individuals over time.

**What this study adds?**

- Pandemic severity increased adherence, while we find no evidence of decline over time (i.e., pandemic fatigue).
- Within‐person changes in response efficacy beliefs were important drivers of changes in behaviour.
- Previous, cross‐sectional research likely overestimated effects of psychosocial factors on behaviour.

## INTRODUCTION

During the COVID‐19 pandemic, governments advised or directed people to change their behaviour to reduce the spread of the virus. In order to find methods to improve adherence to such behavioural measures, many studies have investigated associations between psychosocial factors and protective behaviour (Liang et al., 2022; Moran et al., 2021). Although these studies provided useful first insights, they also had their methodological limitations. First, most studies used data from one specific moment in the pandemic, while the changing pandemic context may play an important role: changing pandemic circumstances could have affected psychosocial factors and behaviour directly (Cipolletta et al., 2022) and may have moderated effects of psychosocial factors on behaviour (Schmitz et al., 2022; Smith et al., 2022). Second, most studies used between‐person analyses for examining what are essentially within‐person psychological processes (Liang et al., 2022; but see Scholz et al., 2023). Third, studies often used generic measures of protective behaviours (encompassing multiple behaviours such as physical distancing, hygienic behaviours and testing and isolation) which can obscure important differences between behaviours (Gibson‐Miller et al., 2022; Wismans et al., 2022).

The current, preregistered, study used unique data from a large‐scale dynamic cohort study conducted throughout the COVID‐19 pandemic in the Netherlands to investigate how contextual as well as (individual) psychosocial factors affected preventive behaviour. With these data, we can study within‐person changes in psychosocial factors and behaviour to better approximate possible causal effects. In line with WHO guidelines on behavioural insights on COVID‐19 as well as many previous studies, psychosocial factors were derived from the extended health belief model and protection motivation theory and included both general (e.g., perceived pandemic threat) as well as behaviour‐specific (e.g., perceived effectiveness of a behaviour) factors (WHO Regional Office for Europe, 2020). We focused on two behaviours that were central in the Dutch Governments' behaviour advice, as well as that of other countries: handwashing (a primarily private and familiar behaviour) and physical distancing (a primarily social interaction and novel behaviour that were expected to require more effort). Because distancing behaviour strongly depends on the social and physical context (Bussemakers, van Dijk, van den Putte, & de Bruin, 2023), we study context‐specific distancing and focus on two contexts that were relevant for almost the entire Dutch public but also strongly differ in their social and physical characteristics: distancing when visiting with friends and family as well as distancing when grocery shopping.

**FIGURE 1:**
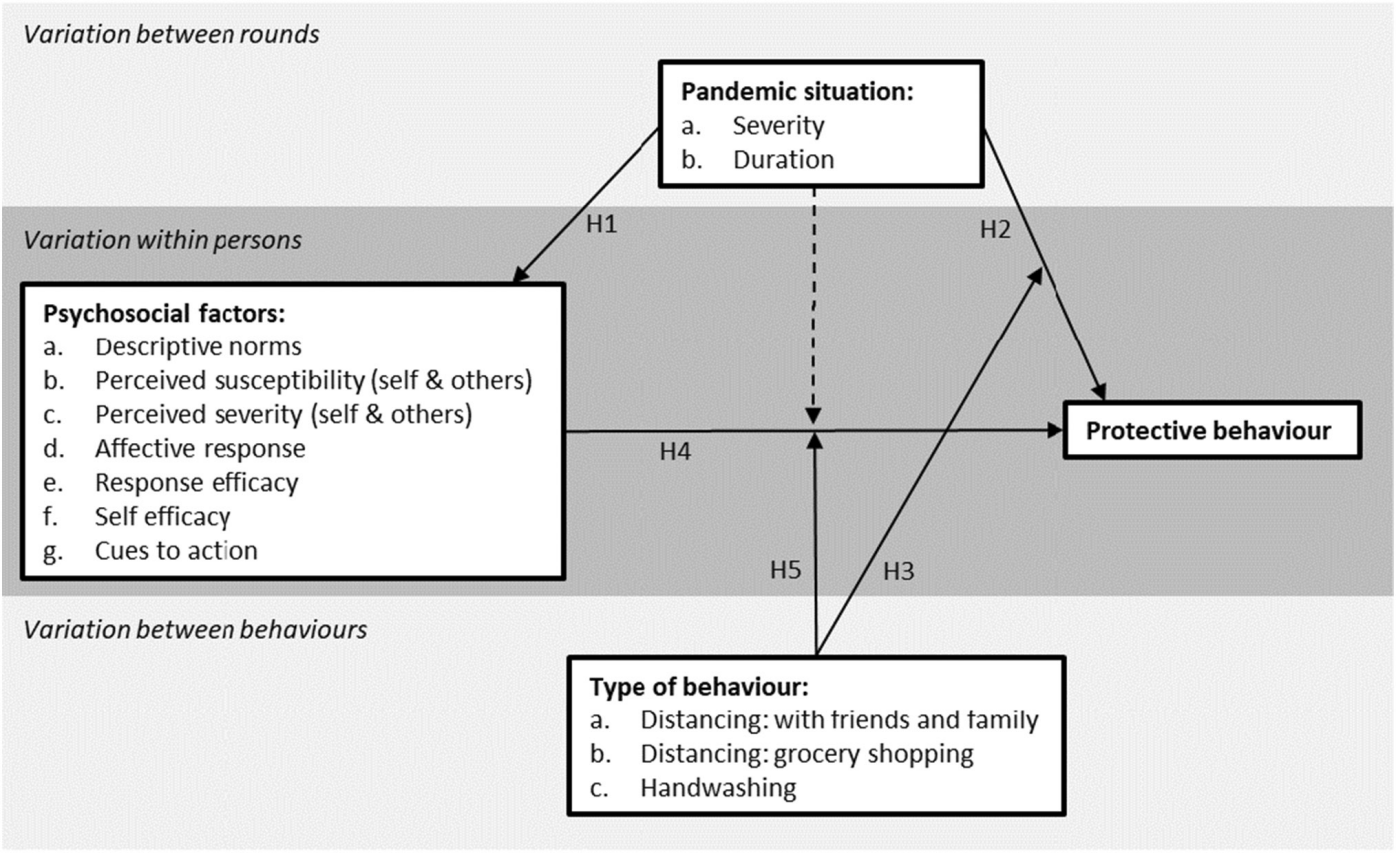
Conceptual model.

In the preregistration, we described a conceptual model of the expected associations based on theory and previous literature (see Figure 1). Regarding the contextual influences, we expected that a more severe COVID‐19 situation would increase average levels of psychosocial factors (H1a) and protective behaviours (H2a) (Schneider et al., 2021). Conversely, we expected that over time, levels of psychosocial factors (H1b) and behaviour (H2b) would reduce due to higher rates of population immunity and possibly also pandemic fatigue, net of changes in pandemic severity (Franzen & Wohner, 2021; Wright et al., 2022). Given the relatively high social costs of distancing, particularly with friends and family, and indications that people may see close contact with friends or family as relatively safe (Burton et al., 2022; De Vries & Lee, 2022), we expected that people were more likely to reduce this behaviour when the pandemic situation was less severe and the pandemic lasted longer. Conversely, handwashing behaviour was expected to have been more stable over time because people were familiar with it prior to the pandemic and it does not come at a high personal or social cost (H3) (Schmitz et al., 2022; Zhang et al., 2022).

Regarding within‐person differences, we expected that all psychosocial factors were positively associated with protective behaviour (H4) (Grano et al., 2022; Herbas‐Torrico & Frank, 2022), but the relative importance of these factors may differ between behaviours. We expected that psychosocial factors of a social nature (descriptive norms, perceived likelihood of infecting others and the perceived severity of doing so) would be more strongly associated with physical distancing from friends and family than with handwashing (H5) (Burton et al., 2022; Schmitz et al., 2022).

Finally, we explored to what extent the severity and duration of the pandemic moderated associations between psychosocial factors and behaviour (dotted arrow in Figure 1). We did not formulate hypotheses on the direction of these moderations, as there was limited previous evidence on this topic (Schmitz et al., 2022; Smith et al., 2022).

## MATERIALS AND METHODS

Preregistration, analysis code and output of this article are available on OSF (https://osf.io/8zjvu/).

### Data

We used individual‐level data from the Corona Behavioural Unit COVID‐19 cohort, a dynamic cohort study conducted between April 2020 and September 2022 among the Dutch population by the National Institute for Public Health and The Environment (RIVM) (van den Boom et al., 2023). The survey was developed in accordance with the WHO toolkit for behavioural insights during the COVID‐19 pandemic (WHO Regional Office for Europe, 2020). We used data up until round 18 for distancing (conducted in January 2022) and round 19 for handwashing (conducted in March 2022) because after these moments, the Dutch Government no longer included these measures in their behavioural policy. We use data from 186,490 unique participants over 971,899 observations (participants in a specific round). Appendix S1 provides descriptive information for all variables and analyses.

### Measures

#### Protective behaviour

Our measure of handwashing behaviour was based on four questions which were found to form a reliable, unidimensional scale (Bussemakers, van Dijk, Dima, & de Bruin, 2023). The questions asked participants how often they washed their hands when leaving their home, when visiting others, before eating and after blowing their nose in the past week on a 7‐point response scale ranging from *never* to *always*. To create the scale score, we calculated the average value of participants' valid answers.

The measures of physical distancing from friends and family, and when grocery shopping were each based on two questions. Participants were first asked how often they visited with friends and family or shopped for groceries in the past 7 days. If they indicated they did so at least once, a follow‐up question asked them how often others came closer than 1.5 m the last time did this activity. This question originally had six categories, but an earlier analysis indicated better reliability after merging them into four categories (Bussemakers, van Dijk, Dima, & de Bruin, 2023). We created two variables reflecting distancing when visiting with friends and family, or when grocery shopping with four response categories:

1. Not in situation
2. Never closer than 1.5 m (original answer: others never closer than 1.5 m)
3. Infrequently closer than 1.5 m (original answers: others seldom/sometimes closer than 1.5 m)
4. Frequently closer than 1.5 m (original answers: others regularly/often/very often/always closer than 1.5 m)

#### Psychosocial factors

We included the following psychosocial factors: risk perception (i.e., perceived susceptibility to and perceived severity of a COVID‐19 infection), self‐efficacy, response efficacy, descriptive social norms and affective response (emotional response and affective risk). Table 1 provides an overview of the questions used to measure each characteristic. Reliability analyses indicated that the factors measured with single items (perceived susceptibility and severity for oneself and others, self‐efficacy, response efficacy and descriptive norms) had sufficient reliability if included categorically (five categories), while the two multi‐item affective response measures formed internally consistent, continuous scales when averaging participants' answers (see Appendix S2 for details).

**TABLE 1:** Measurement of psychosocial factors.

Psychosocial factor	Question(s)	Answer categories
Perceived personal susceptibility	How likely is that you will be infected with the coronavirus in the upcoming months	1. ‘Very unlikely’ to 5. ‘Very likely’
Perceived susceptibility others	Imagine that you are infected with the coronavirus. How likely is it that you will infect others?	1. ‘Very unlikely’ to 5. ‘Very likely’
Perceived personal severity	How bad would it be if you are infected with the coronavirus?	1. ‘Not bad at all’ to 5. ‘Very bad’
Perceived susceptibility others	Imagine that you are infected with the coronavirus. How bad would it be if you infected someone else?	1. ‘Not bad at all’ to 5. ‘Very bad’
Self‐efficacy	How difficult or easy is it for you to regularly wash your hands 20 s with water and soap? How difficult or easy is it for you to always keep 1.5 m distance from others?^a^	1. ‘Very difficult’ to 5. ‘Very easy’
Response efficacy	How well would the following advice help to reduce the spread of infections: regularly wash your hands 20 s with water and soap? How well would the following advice help to reduce the spread of infections: always keep 1.5 m distance from others?^a^	1. ‘Does not help’ to 5. ‘Helps very much’
Descriptive norms	I see that most people around me regularly wash their hands 20 s with water and soap I see that most people around me keep at least 1.5 m distance from others	1. ‘Completely disagree’ to 5. ‘Completely agree’
Emotional response	The coronavirus: Is something I think about all the time/almost never I am very afraid of/not afraid at all I worry about a lot/not at all Makes me feel helpless/does not make me feel helpless Gives me a lot of stress/no stress at all	Participants could indicate their position on a 5‐point scale with the two positions in the questions on the extremes. The other positions were not labelled.
Affective risk	The coronavirus: Feels very close/very distant Spreads very fast/very slow	
Cues to action	COVID‐19 symptoms, current (from round 6 onwards: not due to chronic health problems) COVID‐19 symptoms, past 6 weeks (not due to chronic health problems) Positive test: participant Positive test: household member Positive test: other contact Vaccination status	No vs. yes

^a^ In rounds 6–11 and 14 of the study, situation‐specific questions were included for self‐ and response efficacy (self/response efficacy of distancing when visiting with friends and family and when grocery shopping). Using these measures yielded smaller, non‐significant effects (see Appendix S3). This was likely due to more limited variation of the variables in the analyses compared to the main analyses, as the rounds that included these questions were almost all collected during the second wave of COVID‐19 infections in the Netherlands.

Additionally, we included three types of situations that may have served as cues to action for protective behaviour: having COVID‐19‐related symptoms (currently or in the 6 weeks before the survey was carried out), testing positive for COVID‐19 in the prior 6 weeks (participants themselves, their household members or other contacts) and vaccination status (not vaccinated, first dose, second dose, etc.), the latter expected to have served as a negative cue to action. Cues to action were not available in all rounds: symptoms in the 6 weeks before the survey were asked from round 6 onwards when participants could also indicate whether their symptoms were due to chronic health problems, which were not regarded as COVID‐19 symptoms in that case. Due to this change in measurement, we conducted separate within‐person analyses for the rounds before and after this change. Testing and vaccination questions were included after these became available.^1^ In the rounds when cues to action were not asked, all observations were coded *no* (0, not having experienced this cue to action in that round).

#### Pandemic context

Pandemic context was measured with two indicators: severity and duration of the COVID‐19 pandemic. Severity of the COVID‐19 pandemic was measured using the number of patients with COVID‐19 admitted to hospitals. Admissions were registered daily, but to account for day‐to‐day fluctuations, we calculated a rolling average over the past 7 days prior to each day. Because the behavioural measures referred to behaviour in the week before the survey, we used the value for hospitalizations 1 week before the survey was sent out to participants (thus indicating average hospitalizations 14–7 days before the survey‐date) in the regression models. As a (preregistered) robustness test, we also studied the effect of severity measured by policy stringency because earlier research indicated people also used to stringency of the policy response as an indicator of the threat of the pandemic (Foad et al., 2021). This analysis yielded highly similar results (see Appendix S4).^2^ Duration is measured in number of days since the first COVID‐19 case in the Netherlands (27 February 2020).^3^ Additionally, there were three changes in policy that could affect distancing behaviour which were therefore included as controls: whether keeping 1.5 m distance was strongly advised or obligated, whether there was a maximum number of guests people could receive at home (for distancing when visiting with friends and family) and whether it was strongly advised or obligated to wear a face mask in stores (for distancing when grocery shopping).

### Analytical approach

H1 and H2 on the associations between changes in the pandemic situation and changes in psychosocial factors and behaviour were investigated with longitudinal plots displaying trends in these factors over time. To ease interpretability, we included all categories only for distancing behaviour, while we dichotomized the categorical psychosocial determinants (distinguishing positive (*4/5*) responses from neutral or negative responses (*1/2/3*)). Furthermore, we tested the associations using regression models with contextual factors as predictors of average levels of psychosocial factors and behaviour (using *p* < .05). To account for possible ****changes in the cohort composition, we modelled average levels of behaviour and determinants in multilevel models (observations nested in participants) that controlled for sociodemographic characteristics.^4^^,^ 5 To investigate H3, we compared the trends and strength of associations between the three outcome variables.

To investigate H4 and H5, we used fixed effects (FE) models, which estimate within‐person effects that are independent of time‐invariant differences between participants (Bell et al., 2018; Bell & Jones, 2014). These models therefore offer a stricter test of the impact of psychosocial factors on behaviour than cross‐sectional or longitudinal random effects models, although estimates may still be biased by unobserved time‐variant characteristics. Because the measure of having symptoms that may be caused by COVID‐19 was changed in round 6, we used separate models for data collected before and after this round.

Given our large N at the individual level, even very small effects can be statistically significant, so we also considered effect size. For the continuous outcome measure (handwashing), effect sizes were calculated by dividing the estimated effects of psychosocial factors by the within variation of handwashing behaviour (Kittel et al., 2021). In line with common interpretations of effect sizes in the psychological literature, we considered effects <.2*sd* very small, between .2*sd* and .41*sd* small, between .41*sd* and .63*sd* moderate and >.63*sd* large (Ben‐Sachar et al., n.d.; Gignac & Szodorai, 2016). We are not aware of any agreed‐upon effect sizes for average marginal effects, so we aligned them with the effect sizes used for handwashing. Because these are based on standard deviations of normally distributed outcome variables which are two sided, while categorical variables are not, we split the cut‐off values in two and consider effects <10% very small, effects between 10% and 20% small, between 20% and 31% moderate and >31% large. When interpreting effects, we focus on effects that are both statistically significant (*p* < .05) and at least *small* in terms of effect size.

All psychosocial factors measured at t‐1 were included as predictors in these models. Lagged effects ensured that the impact of psychosocial factors on behaviour in the week before the survey was estimated and not the reversed effect. For cues to action, we also explored longer time lags and found that including lags up to four rounds was most appropriate to estimate the impact of participants or their household members testing positive, while no additional lags were necessary for the other cues.^6^ We used OLS regression for handwashing and logistic regression for distancing behaviour. For the logistic models, we converted effects to average marginal effects as these can be interpreted as percentual changes and are comparable across models, unlike odds ratio (Mood, 2010). Originally, we planned to use *never within 1.5 m from others* as the reference category for the analysis of distancing behaviour, but we changed this to *frequently* as the trend analyses indicated changes in this category (compared to *infrequently*) to be most relevant. For H5 on differences in the relative importance of specific psychosocial factors for each type of behaviour, we compared the relative size of the effects of the different psychosocial factors with each other.

Finally, to investigate whether associations between psychosocial factors and behaviour varied with the pandemic situation, we added the contextual variables as well as the interaction between these contextual characteristics and psychosocial factors to the FE models (separate models for severity and duration). Unfortunately, this was not possible for rounds 1–6, as these have insufficient variation in pandemic context factors to investigate how these affect the impact of psychosocial characteristics. Furthermore, collinearity made it impossible to reliably estimate interactions for vaccination with pandemic duration for both outcomes (as vaccine availability was time specific), and positive tests with both contextual characteristics for distancing behaviour.

## RESULTS

### Trend analyses of behaviour and determinants (H1, H2 and H3)

**FIGURE 2:**
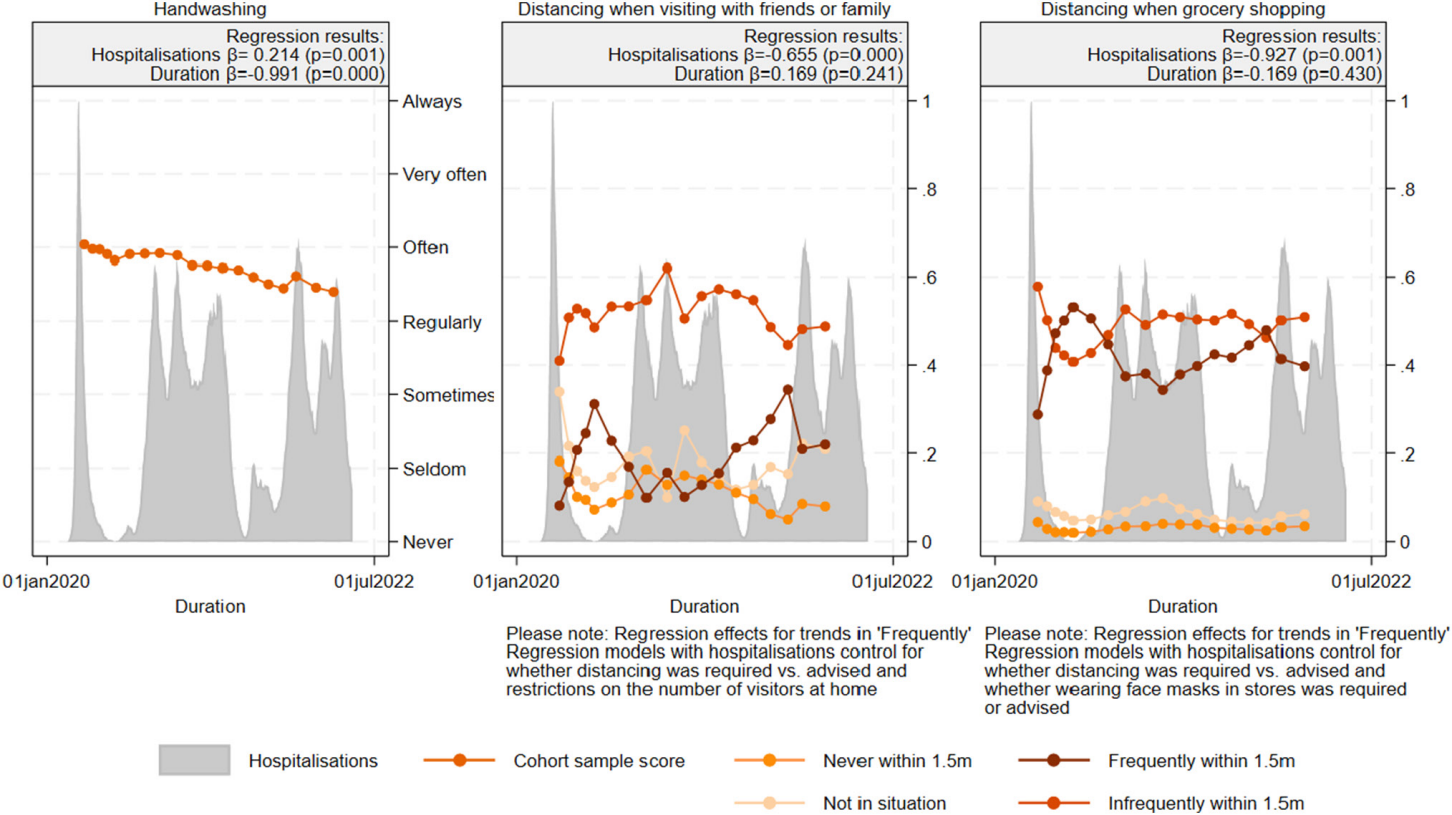
Behaviour (cohort average) with severity and duration of the pandemic.

Figure 2 displays how average handwashing and distancing behaviour changed with the severity and duration of the pandemic, with the results of the regression models displayed on top of the figure. For determinants, Table 2 includes the regression coefficients of severity and duration, while figures for trends in determinants as well as the full regression models can be found in Appendix S4. In line with hypothesis 2a, we found that adherence to behavioural guidelines clearly increased when the pandemic situation became more severe. In line with hypothesis H3, handwashing was the most stable type of behaviour and least affected by pandemic severity, as shown by the smaller increases in trends when hospitalizations increased. However, contrary to H3, changes in distancing when grocery shopping were affected more by changes in pandemic severity than changes in distancing from friends and family. Table 2 shows that all psychosocial determinants except the indicators related to risk perception (perceived susceptibility to and perceived severity of infection) and response efficacy of handwashing increased with the severity of the pandemic situation, largely confirming hypothesis 1a.

**TABLE 2:** Regression results of psychosocial determinants (cohort average) on severity and duration.

	Perceived susceptibility—self	Perceived susceptibility—others	Perceived severity—self	Perceived severity—others	Emotional response	Affective risk
Duration: days since start of pandemic	.434	−.442	−.581*	−.883***	−.681***	.272
Hospital admissions (7‐day average) lagged (7 days)	.302	.307	.133	.099	.518**	.637**
Observations	19	19	19	19	19	19

	Self‐efficacy—handwashing	Self‐efficacy—distancing	Response efficacy—handwashing	Response efficacy—distancing	Descriptive norms—handwashing	Descriptive norms—distancing
Duration: days since start of pandemic	−.903***	−.577***	−.975***	−.627**	−.953***	−.737***
Hospital admissions (7‐day average) lagged (7 days)	.269*	.768***	.060	.516**	.179*	.473**
Observations	19	19	19	19	18	18

*Note*: Standardized beta coefficients.

*p < .05, **p < .01, ***p < .001.

Regarding the impact of the duration of the pandemic, changes were smaller than we expected. Contrary to H2a, Figure 2 only shows a decreasing trend for handwashing over time, not for distancing. This is surprising as we expected stronger reductions in distancing over time due to high social costs of this behaviour (H3). In line with hypothesis 1b, Table 2 shows that perceived severity, emotional response, self‐efficacy, response efficacy and descriptive norms also decreased over time.

### Fixed effects models of determinants and adherence (H4 and H5)

**FIGURE 3:**
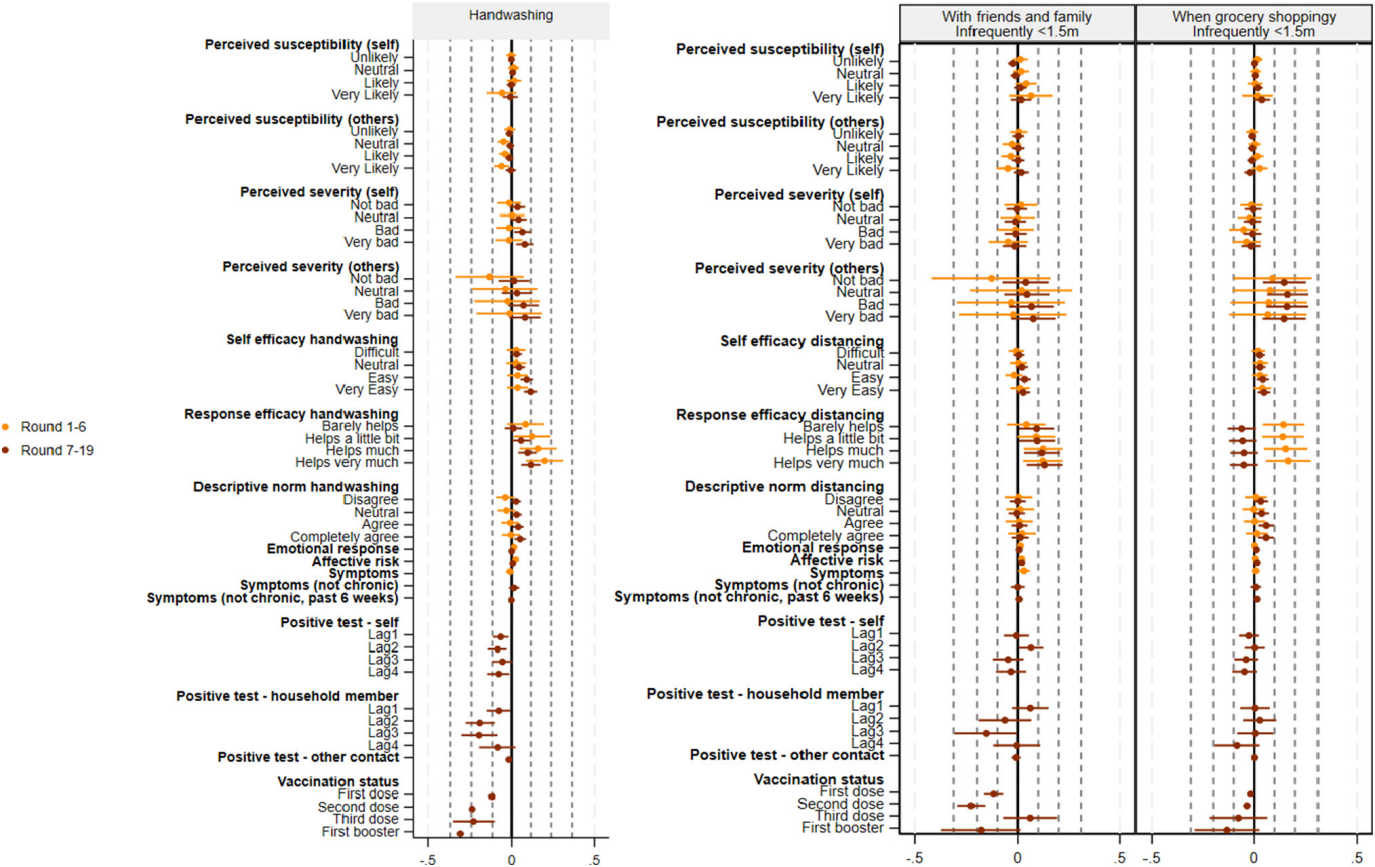
FE associations between psychosocial determinants and behaviour (marginal effects for distancing, relative to ‘frequently <1.5m from others’).

Figure 3 presents the results of the FE models of associations between psychosocial determinants (lagged) and handwashing behaviour and infrequently (vs. frequently) being within 1.5 m from others. Full results for the models of distancing can be found in Appendix S5. The figure includes 95% CIs and the dotted lines indicate values for small, moderate and large effects. It shows that contrary to H4, most within‐subject associations between psychosocial determinants and behaviour were very small and/or not statistically significant. Only three factors consistently affected behaviour. First, response efficacy had a small effect on both handwashing and distancing behaviour: when participants' perceived efficacy of handwashing and distancing had increased in the prior round, they more often washed their hands and were more likely to be infrequently (instead of frequently) close to others when visiting with their friends and family or when grocery shopping (although the latter was only found in the analyses of round 1–6). Second, vaccination had a substantial effect on all three outcomes, reducing both handwashing as well as distancing. Third, although we expected that positive COVID‐19 tests would serve as a cue to action, our results indicated that they had the opposite effect: having a household member who tested positive reduced handwashing behaviour in the rounds after and people who tested positive or who had a household member who tested positive were less likely to avoid grocery shopping in later rounds. Some other determinants were found to affect specific types of behaviour in rounds 7–19, but not in rounds 1–6. We found a small effect of self‐efficacy on handwashing behaviour and an increase in the perceived severity of infecting others was associated with more distancing when grocery shopping. However, our findings contradicted H5 on the relative importance of psychosocial factors of a social nature as we did not find relatively stronger effects of descriptive norms and the perceived likelihood and severity of infecting others for distancing, particularly when visiting with friends and family.

Regarding the moderating impact of contextual factors on individual‐level effects, we found that most psychosocial determinants had very similar effects at higher and lower levels of pandemic severity and duration (results can be found in Appendix S6). Although we found a few substantial and statistically significant differences in effects of psychosocial factors depending on the pandemic context, these did not show a consistent pattern (e.g., in a less severe pandemic situation, the second vaccination dose had a stronger negative effect on distancing from friends and family, while the third dose had a stronger positive effect on the same outcome). We cannot rule out that these significant differences simply resulted from chance, given the large number of estimated interactions and inconsistent patterns.

### Additional analyses

We found smaller effects of psychosocial factors on behaviour than previous studies, which could be due to our use of within‐person, lagged effects from multiple regression models. Others have looked primarily at cross‐sectional between‐person effects, even though the psychological theories concern within‐person processes in psychology that would affect behaviour. To check whether this might indeed explain these differences, we estimated concurrent rather than lagged within‐person associations between determinants and behaviour (preregistered) as well as between‐subject differences (explorative and for handwashing only, as between‐within models can only be reliably estimated for continuous outcome variables (Allisson, 2014)). Direction and effect sizes of these models are presented in Table 3, and full model results can be found in Appendices S7 and  S8. The main observation from these models is that associations between psychosocial factors and handwashing behaviour were considerably larger in the concurrent and between‐subject models. However, for distancing behaviour, the concurrent analyses for distancing did not yield larger effects. Overall, these findings support the notion that effects of psychosocial determinants have been overestimated in previous studies due to their cross‐sectional design.

**TABLE 3:** Overview of the direction and effect size of the estimated effects of psychosocial determinants on behaviour per model.

	Main models (lagged, within)						Determinants and behaviour measured concurrently (within)						Between‐person associations (lagged)		Determinants included separately (lagged, within)					
	Handwashing		Distance—friends & family		Distance grocery shopping		Handwashing		Distance—friends & family		Distance—grocery shopping		Handwashing		Handwashing		Distance—friends & family		Distance—grocery shopping	
Perceived susceptibility (self)													+	Small			+	Small		
Perceived susceptibility (others)													−	Small						
Perceived severity (self)							−	Small					+	Small	+	Small /Mod.	+	Small		
Perceived severity (others)					+	Small	+	Small /Mod.					+	Small/Mod.	+	Mod./Large	+	Small/Mod.	+	Small
Self‐efficacy	+	Small					+	Mod./Large					+	Large	+	Small/Mod.	+	Small	+	Small
Response efficacy	+	Small	+	Small	+	Small	+	Mod./Large			+	Small	+	Mod./Large	+	Mod./Large	+	Mod./Large	+	Small/Mod.
Descriptive norms							+	Small/Mod.			+	Small	+	Small/Mod.	+	Small	+	Small	+	Small
Emotional response																				
Affective risk																				
Symptoms							+	Small					n.a.	n.a.						
Symptoms (not chronic)													n.a.	n.a.						
Symptoms (not chronic, past 6 wks)													n.a.	n.a.						
Positive test (self)					−	Small/Mod.	+	Small					n.a.	n.a.	−	Small	−	Small	−	Small
Positive test (household member)	−	Small			−	Small/Mod.	+	Small					n.a.	n.a.	−	Small/Mod.	−	Mod.	−	Small
Positive test (other contact)													n.a.	n.a.						
Vaccination status	−	Small/Mod.	−	Small/Mod.	−	Small	−	Small/Mod.					n.a.	n.a.	−	Small/Mod.	−	Small/Mod.	−	Small

*Note*: Empty cells indicate effects were not statistically significant and/or did not meet the criteria for an effect size that was at least small.

Furthermore, we employed multiple regression techniques to account for possible confounding influences in our main models. Crutzen and Peters (2023) argued that such models could underestimate associations, as psychosocial determinants conceptually overlap and may mediate each other. Bivariate analyses could therefore point to additional factors that influence behaviour through mediating processes or overlap with factors that are shown to affect behaviour in multivariate models. Indeed, including psychosocial determinants separately in the models yielded considerably stronger effects on all three outcomes, as shown in Table 3 (full model results can be found in Appendix S9): changes in perceived severity of COVID‐19 infections, self‐efficacy and descriptive norms were also associated with changes in behaviour, while associates of efficacy beliefs became larger (self‐efficacy for handwashing and response efficacy for all behaviours). Because the latter also had substantial effects in the multivariate models, these results indicate that perceived severity, self‐efficacy and descriptive norms affected behaviour indirectly through response efficacy (and self‐efficacy for handwashing), although we cannot determine the exact process underlying these indirect effects (mediation, conceptual overlap or both).

## DISCUSSION

Our study examined whether changes in pandemic context and psychosocial factors affected changes in people's handwashing and distancing behaviour during the COVID‐19 pandemic, and the degree to which these results differ from the cross‐sectional results typically presented in this literature. Regarding the contextual factors, pandemic severity was very clearly associated with higher levels of most psychosocial factors as well as distancing behaviour and, to a somewhat lesser extent, handwashing. Although distancing from friends and family varied most over time, the influence of pandemic severity was relatively smaller for distancing from friends and family compared to distancing when grocery shopping. This may indicate behavioural measures aimed at public spaces such as crowd reduction were more influential than measures aimed at private settings such as limiting the number of visitors at home (Liebst et al., 2021). Moreover, in private circumstances also other factors play a role, such as the need for close social contact (Burton et al., 2022). Pandemic fatigue appeared not to play a role, as we did not find that people were less likely to distance themselves from others as the pandemic progressed. We found a small decline in handwashing over time, which could have been due to the relatively strong reduction in its perceived efficacy following widespread media coverage of research showing that handwashing was less useful than initially communicated by the government. Overall, the small decline in handwashing and the lack of decline in distancing contradict previous studies finding that pandemic fatigue led to lower adherence to behavioural guidelines over time (Franzen & Wohner, 2021; Ross & Dutton, 2023). This difference is likely due to our inclusion of pandemic severity in the models, which means we could provide a better estimate of the duration effect (Wilson, 2023). Our study expands on earlier analyses that indicated that support for measures remained high throughout the first year of the pandemic in the Netherlands (De Wit et al., 2023), showing that even with prolonged duration, people increased their adherence to behavioural guidelines when the pandemic situation became more severe.

At the individual‐level, within‐person multivariable analyses indicated that response efficacy was most relevant for both handwashing and distancing behaviour, while self‐efficacy and perceived severity of an infection had independent effects on, respectively, handwashing or distancing when grocery shopping. Moreover, bivariate within‐person analyses indicated self‐efficacy, perceived severity and social norms were also associated with behaviour, indicating they could indirectly affect behaviour. The importance of response efficacy is in line with a review and meta‐analysis of mostly cross‐sectional studies by Liang et al. (2022), as well as a recent longitudinal study (Scholz et al., 2023), which both found that response efficacy was an important driver of protective behaviour. Our findings also nuance previous results: whereas previous cross‐sectional research highlighted the importance of self‐efficacy and social norms (Dixon et al., 2022; Liang et al., 2022), we only find a direct, small association between self‐efficacy and handwashing, while descriptive norms only had an indirect effect in our within‐person models. Earlier cross‐sectional associations between descriptive norms and behaviour may have been overestimated due to social selection: when some groups such as people with a higher socioeconomic status are more likely to adhere to (health) behavioural guidelines, partly due to factors such as higher efficacy beliefs, they will tend to report both higher adherence as well as related descriptive norms (Bish & Michie, 2010; Mercer & Mollborn, 2023). This, in turn, could create a large, mostly spurious association between descriptive norms and behaviour.

Although we found a clear effect of pandemic severity at the contextual level, individual factors related to risk perception had a small, mostly indirect impact and emotional and affective responses were not associated with behaviour. Previous research showed that people's support for strict behavioural policies was guided by the general threat of COVID‐19, rather than the perceived threat to themselves (Foad et al., 2021). This could also explain the relatively large impact of response efficacy, which was measured by asking people how effective measures were in order to reduce the spread of COVID‐19, rather than the effectiveness of these measures for protecting oneself from infection.

In a similar vein, cues to action also had a substantial impact on behaviour: when having symptoms or immediately after a positive test, people washed their hands more often, but in the longer term both positive tests and vaccination reduced people's adherence to behavioural guidelines. This indicates people acted quite rationally and altruistically: more careful in the short term due to increased chances of infecting others (Elazab et al., 2023), and less in the longer term because previous infections or vaccinations reduced COVID‐19 risks.

Finally, we did not find consistent moderating effects of the pandemic context on the impact of individual psychosocial factors on behaviour. Associations between psychosocial determinants and behaviour did not change with the severity or duration of the pandemic, supporting studies that found similar associations between determinants and behaviour at different time points (Schmitz et al., 2022; Scholz et al., 2023; Smith et al., 2022). The impact of determinants varied more between behaviours, as we found that psychosocial characteristics were more relevant for handwashing, an established and mostly private behaviour than for distancing, particularly distancing from friends and family. We therefore recommend future research to study people's motivations for distancing in social situations in particular.

The cohort data used in this study allowed for longitudinal analyses, but they also had their limitations. Most importantly, the 6‐week span between survey rounds was relatively long, especially considering changing pandemic circumstances. This could have suppressed the lagged effects between psychosocial factors and behaviour and also means the data were less suitable to study associations among determinants and how they jointly influence behaviour. Our findings indicate that some psychosocial factors such as social norms affect behaviour through response efficacy, but the exact nature of these mechanisms remains unclear. Future research might disentangle the relationships between these factors and how they jointly influence behaviour using data collected over a shorter time span, although this may come at the cost of a smaller, more selective sample. Furthermore, although our distancing measures distinguish situations such as grocery shopping and visiting with friends and family, there could still be variability within these settings. To improve recollection, participants were asked to report their distancing behaviour during the last time they were in a specific situation in the past week, but these situations may differ between survey rounds (e.g., once a meeting with a younger friend and another time an elderly family member). Such differences would result in arbitrary within variation in behaviour, reducing the explanatory power of the psychosocial constructs. Finally, although our study included various psychosocial characteristics including risk perceptions, efficacy beliefs, descriptive norms, as well as measures related to the pandemic context, these factors of course did not fully predict all variation in protective behaviour over time. Future longitudinal research might therefore include other relevant factors such as the (social) costs of behaviour, injunctive social norms and/or moral norms. Regarding the role of the pandemic context, future research might investigate how people's response to pandemic severity changes over time or depends on congruence versus disagreement between pandemic severity and political response (Martin‐Lapoirie et al., 2023; Waterschoot et al., 2023).

Our study highlights the usefulness of longitudinal data and within‐person models to detect possible causal associations in behavioural research. The fact that we find smaller associations in our primary within‐person longitudinal models than in our between‐person cross‐sectional models indicates that effects of psychosocial factors have been overestimated in previous cross‐sectional research. An additional advantage of these models is that we can illustrate temporal effects of time‐specific factors, such as the differential short‐ and long‐term effects of positive tests on behaviour change.

Our results also demonstrate that people continued to increase adherence to behavioural guidelines when the pandemic situation became more severe, even during later stages of the pandemic. At the individual level, our results show the importance of response efficacy as well as self‐efficacy, perceived severity of infecting others and descriptive norms which affected behaviour indirectly. This supports previous findings outside of the pandemic context, suggesting emphasizing the feasibility and effectiveness of behaviours in reducing health risks such as COVID‐19, rather than focusing on fear arousal (Ruiter et al., 2014; Scholz et al., 2023; Travaini et al., 2023).

## AUTHOR CONTRIBUTIONS

**Carlijn Bussemakers:** Conceptualization; methodology; formal analysis; data curation; writing – original draft; writing – review and editing; visualization; project administration. **Nicole Stappers:** Conceptualization; methodology; writing – review and editing. **Floor Kroese:** Conceptualization; methodology; writing – review and editing; resources. **Bas van den Putte:** Conceptualization; methodology; writing – review and editing; resources. **Marijn de Bruin:** Conceptualization; methodology; resources; writing – review and editing; project administration; supervision; funding acquisition.

## FUNDING INFORMATION

The Corona Behavioural Unit initiative has been made possible by funding from ZonMW (Netherlands Organization for Health Research and Development) and the Dutch Ministry of Health, Welfare and Sport. The funders of the study had no role in the study design, data collection, data analysis, data interpretation or writing of the article.

## CONFLICT OF INTEREST STATEMENT

The authors report no conflicts of interest.

## ETHICAL STATEMENT

The cohort study was exempted from formal ethical review by the Centre for Clinical Expertise at the National Institute of Public Health and the Environment (RIVM) (Study Number G&M‐561) as it does not meet the requirement as laid down in the Law for Research Involving Human Subjects (WMO). Informed consent was provided by all participants in each round. Data collection was outsourced to a research agency (‘Research 2Evolve’).

## Supporting information


Appendices S1‐S9.

## Data Availability

Due to European privacy regulation (GDPR, or General Data Protection Regulation), data cannot be shared publicly, unless aggregated. For academic collaborations and publishing in scientific journals, the RIVM initiated a Behavioural Science Consortium (BePrepared), so that collaborating researchers working at various universities can access the data in the secured environment of the RIVM. Preregistration, analysis code and output of this article are available on OSF (https://osf.io/8zjvu).

## Footnotes

^1^
Whether participants tested positive for COVID‐19 in the 6 weeks prior to the survey was available from round 7 onwards. Whether a household member tested positive was asked from rounds 8 until 13 as well as rounds 17 to 19. Whether participants had been in contact with another person who tested positive was available from rounds 8 to 19. In rounds 6 and 7, the question asked whether participants were informed about such a contact by the regional public health service, from round 8 onwards other information channels (e.g., the infected person themselves or the Covid tracker app) were included. Finally, vaccination status was asked after the onset of mass vaccination campaigns, but because some participants, such as healthcare professionals, received vaccinations early at the start of these campaigns, the questionnaires of rounds 14–18 also asked when participants received their vaccinations.

^2^
We also considered including the number of infections as an indicator of pandemic severity, but unfortunately, there was no reliable measure of the number of infections for the entire period under study. The number of positive tests only serves as a useful indicator of infections for the period when large‐scale testing was implemented and self‐tests were not yet available, while measures such as sewage monitoring only became available later in the pandemic. We also considered ICU admittance but decided not to include it as this is an indirect indicator of pandemic severity: not only is there a considerable time lag between changes in infections and ICU admittance but the association between the two also changed depending on the type of medical treatment available in hospitals as well as changing hospital policies.

^3^
We also planned to include duration squared to account for possible non‐linear effects, but this was not possible due to multicollinearity with duration (for all outcome variables).

^4^
In line with previous analyses on the associations between sociodemographic factors and behaviour (Bussemakers et al., 2023), we included the following sociodemographic control variables: sex, age, education level, country of origin, living situation, underlying health problems, employment status, being in education, not being able to keep distance due to work or care responsibilities, urbanization and socioeconomic status of the municipality participants lived in.

^5^
In testing hypotheses H1a and H1b, we focus on all psychosocial factors except cues to action. The cues to action included in this article (positive COVID‐19 tests, vaccination) are inherently linked to the severity and duration of the pandemic, so analysing these relationships offers little substantive insights. Moreover, analysing trends in cumulative states such as vaccination requires a different modelling approach. Given this additional complexity and that the models provide little information, we decided to leave out these analyses.

^6^
Because cues to action related to a specific time point (e.g., a participant tested positive for COVID‐19 between two specific rounds), using a lagged predictor in an FE model to assess the impact assumes that a cue affects behaviour measured at *t*, but no longer at *t* + 1 (when participants are assumed to have returned to the same state as before the cue to action occurred). This is an unrealistic assumption, as cues could still affect behaviour at *t* + 1 and following rounds. We therefore explored how long cues affect behaviour by adding cues from earlier rounds (*t* − 2, *t* − 3 … *t* − n) as predictors.
