# Supplementary material for: Psychosocial determinants of handwashing and physical distancing behaviour during the COVID‐19 pandemic in the Netherlands: A longitudinal analysis
Source: Br J Health Psychol. 2024 Oct 2;30(1):e12755. doi: 10.1111/bjhp.12755 (PMC11586808; doi:10.1111/bjhp.12755)
Supplement: Supplementary file 1 — Appendices S1‐S9. [file BJHP-30-0-s001.docx]

**Appendix S1: Descriptive Statistics**

To study trends in psychosocial factors (H1) and behaviour (H2 and H3), we included all observations (participants in a specific round) with valid information on all sociodemographic characteristics as well as the relevant outcome variable. Table A1 provides the descriptive statistics at the observation-level for these variables.

| **Table A1: Descriptive statistics for the trend analyses (H1-H3) (rounds 1-19)** | | | | | | |
| --- | --- | --- | --- | --- | --- | --- |
|  | N | % | Mean | S.D. | Min | Max |
| *Behaviour* |  |  |  |  |  |  |
| Distancing when visiting with friends or family | 779,686 |  |  |  |  |  |
| Not in situation | 144,205 | 18.5 |  |  |  |  |
| Never closer than 1.5m | 84,510 | 10.84 |  |  |  |  |
| Infrequently closer than 1.5m | 373,635 | 47.92 |  |  |  |  |
| Frequently closer than 1.5m | 177,336 | 22.74 |  |  |  |  |
| Distancing when grocery shopping | 813,588 |  |  |  |  |  |
| Not in situation | 66,493 | 8.17 |  |  |  |  |
| Never closer than 1.5m | 23,511 | 2.89 |  |  |  |  |
| Infrequently closer than 1.5m | 356,919 | 43.87 |  |  |  |  |
| Frequently closer than 1.5m | 366,665 | 45.07 |  |  |  |  |
| Handwashing | 849,273 |  | 4.63 | 1.5 | 1 | 7 |
|  |  |  |  |  |  |  |
| *Psychosocial factors (continuous)* |  |  |  |  |  |  |
| Emotional response | 338,387 |  | 2.87 | 0.9 | 1 | 5 |
| Affective risk | 338,387 |  | 3.55 | 1 | 1 | 5 |
|  |  |  |  |  |  |  |
| *Psychosocial factors (categorical)* |  |  |  |  |  |  |
| Perceived susceptibility (self) | 338,388 | 14.04 |  |  |  |  |
| Perceived susceptibility (others) | 338,388 | 46.30 |  |  |  |  |
| Perceived severity (self) | 338,388 | 55.06 |  |  |  |  |
| Perceived severity (others) | 338,388 | 89.21 |  |  |  |  |
| Self-efficacy - distancing | 335,988 | 63.50 |  |  |  |  |
| Self-efficacy - handwashing | 334,710 | 48.84 |  |  |  |  |
| Response efficacy - distancing | 338,388 | 69.10 |  |  |  |  |
| Response efficacy - handwashing | 338,387 | 80.87 |  |  |  |  |
| Descriptive norms - distancing | 268,184 | 44.97 |  |  |  |  |
| Descriptive norms - handwashing | 309,463 | 60.51 |  |  |  |  |

To study within-person associations between psychosocial factors and behaviour (H4 and H5), we included participants who reported behaviour at *t* as well as psychosocial factors at *t-1* at least twice. Table A2 provides descriptive statistics for the within-person analysis of handwashing, while Table A3 provides this information for distancing behaviour. Because distancing behaviour was measured categorically, reported within-variation requires some explanation: the within percentages per category indicate the percentage of rounds participants were in a specific category conditional on ever being in this category, while the within percentage of the entire variable provides the weighted average of these shares per category, indicating overall levels of stability.

| **Table A2: Descriptive statistics of handwashing behaviour for the within-person analyses (H4-H5) (rounds 1-19)** | | | | | |
| --- | --- | --- | --- | --- | --- |
|  | N | Mean | S.D. | Min | Max |
| Handwashing round 2-6 |  |  |  |  |  |
| Overall | 53,343 | 4.71 | 1.48 | 1.00 | 7.00 |
| Within participants | 17,756 | 0.00 | 0.54 | -5.53 | 4.19 |
| Handwashing round 7-19 |  |  |  |  |  |
| Overall | 143,153 | 4.55 | 1.51 | 1.00 | 7.00 |
| Within participants | 22,882 | 0.00 | 0.62 | -3.90 | 4.78 |

| **Table A3: Descriptive statistics of distancing behaviour for the within-person analyses (H4-H5) (rounds 1-18)** | | | | |
| --- | --- | --- | --- | --- |
|  | **Overall** | | **Within** | |
|  | N | % | N | % |
| Distancing when visiting with friends or family - round 2-6 | 53,359 |  | 17,758 | 59.44 |
| Not in situation | 8,753 | 16.40 |  | 51.59 |
| Never closer than 1.5m | 4,859 | 9.11 |  | 44.82 |
| Infrequently closer than 1.5m | 25,210 | 47.25 |  | 65.76 |
| Frequently closer than 1.5m | 14,537 | 27.24 |  | 61.16 |
|  |  |  |  |  |
| Distancing when visiting with friends or family - round 7-18 | 125,654 |  | 22,670 | 49.14 |
| Not in situation | 24,322 | 19.36 |  | 42.15 |
| Never closer than 1.5m | 13,934 | 11.09 |  | 33.93 |
| Infrequently closer than 1.5m | 62,815 | 49.99 |  | 61.28 |
| Frequently closer than 1.5m | 24,583 | 19.56 |  | 45.31 |
|  |  |  |  |  |
| Distancing when grocery shopping - round 2-6 | 53,359 |  | 17,758 | 68.30 |
| Not in situation | 3,826 | 7.17 |  | 54.15 |
| Never closer than 1.5m | 1,101 | 2.06 |  | 40.48 |
| Infrequently closer than 1.5m | 21,446 | 40.19 |  | 66.88 |
| Frequently closer than 1.5m | 26,986 | 50.57 |  | 74.23 |
|  |  |  |  |  |
| Distancing when grocery shopping - round 7-18 | 133,534 |  | 22,837 | 56.90 |
| Not in situation | 10,936 | 8.19 |  | 38.32 |
| Never closer than 1.5m | 4,261 | 3.19 |  | 27.25 |
| Infrequently closer than 1.5m | 63,986 | 47.92 |  | 62.66 |
| Frequently closer than 1.5m | 54,351 | 40.70 |  | 61.61 |

Some observations lacked a valid answer to the questions on self-efficacy, response efficacy, descriptive norms, or a cue to action (in total, 28.61% of observations have between 1 and 4 missing values on the psychosocial factors, mostly on descriptive norms). We added a separate missing category for these variables in order to retain these participants for the within-analyses.

**Appendix S2: Reliability of the single-item measures of psychosocial constructs**

Most psychosocial factors were measured with single items to limit the length of the survey. A disadvantage of using single-items to measure psychosocial constructs is that the measures may be less reliable than multiple-item measures. We therefore explored the test-retest reliability of all single-items (except for cues to action, as stability herein is not expected). We followed the same method used earlier to investigate the reliability of the behavioural questions, which tested stability in self-reports from round 9 and 10 of the study (winter 2021).^[[1]](#footnote-1)^ This period was chosen because it was relatively stable period in terms of the Covid-19 situation: preventive measures against Covid-19 had been in place for a while and both rounds were conducted during the second lockdown in the Netherlands. For the items measuring perceived susceptibility, perceived severity, self-efficacy and response efficacy, we found that (weighted) kappa>0.4, indicating sufficient reliability for ordinal self-reported measures.^[[2]](#footnote-2)^ For descriptive social norms on adherence to distancing guidelines, we found kappa=0.375, which was just below the 0.4 threshold. We expected that this somewhat lower value, compared to the other measures, reflected an actual change in social norms due to the continuation of the lockdown and increased need for social contact, rather than an indication that of non-reliability of the item. ICC, a measure for stability of continuous variables, was not sufficient (<0.7) for most items, indicating the variables should not be considered continuous.

For the six items related to affective response, factor analyses indicated that the items measured two factors. One factor measured emotional response: four questions strongly loaded on this factor (>0.6) and Cronbach’s alpha of this factor was >0.85. Emotional response scores were therefore calculated by averaging participants’ answers on these four questions. The second factor measured affective risk, but factor loadings (>0.4) and correlations (between 0.43 and 0.6) were lower than for the emotional response factor. Despite these limitations, we averaged participants’ answers on the two questions to measure affective risk, since the combination of the two items likely provides a more reliable measure than the individual two items. A robustness test was conducted using the two separate questions, which showed that fixed-effects associations between affective risk and the three behaviours under study were the same for the single and combined measures. Emotional response and affective risk scores were mirrored so higher scores indicated stronger responses.

**Appendix S3: FE-associations between situation specific efficacy of distancing and distancing behaviour (based on full models, rounds 5-12 and 15)**

**
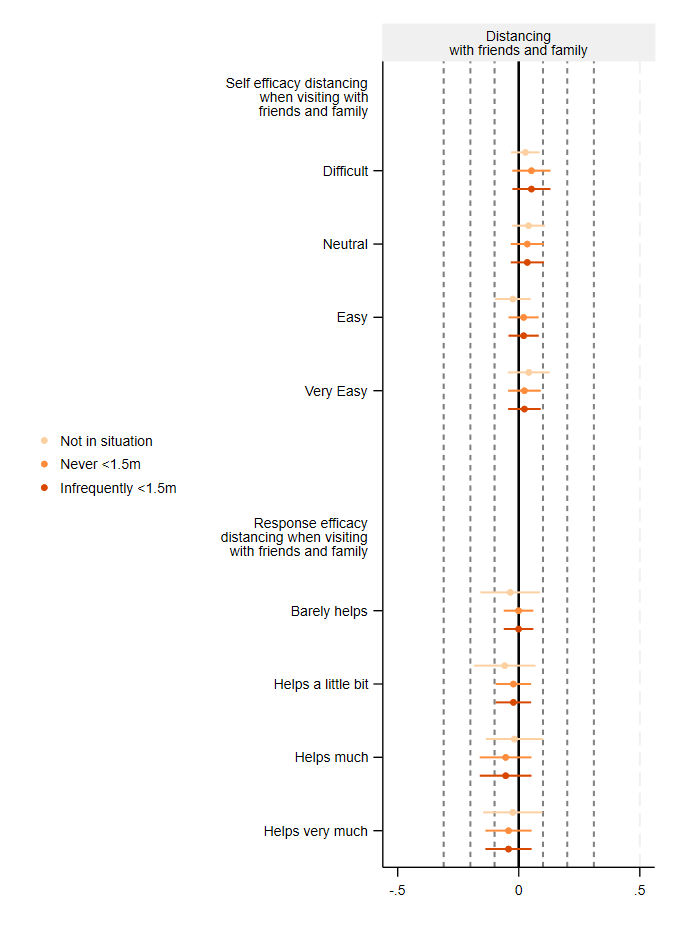

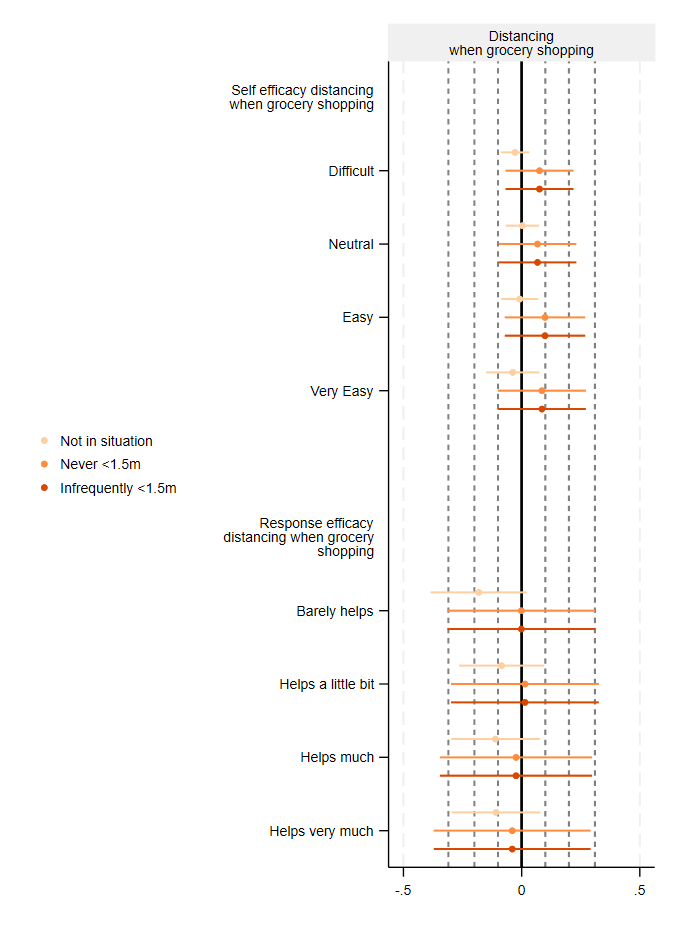
**

**Appendix S4: Trend analyses of behaviour and determinants (H1, H2 and H3) using different operationalisations of pandemic severity**

As a preregistered robustness test, we also operationalized pandemic severity using policy stringency. Results are presented in Appendix S4.1 and S4.2 below.

Additionally, we explored whether measures of severity 1-2 weeks after participants’ behaviour were associated with their behaviour, because the Dutch pandemic communication was mainly based on the number of infections, which preceded hospital admissions and policy changes. Because behaviour referred to behaviour in the week before the survey, we used measures of severity 1 week after the survey took place (for hospitalisations, this was measured with average admissions 1 and 2 weeks after the survey to encompass the entire relevant week). Results can be found in Appendix S4.3 below and indicate that for hospital admissions, results were similar. For stringency, we found that future stringency tended to be more strongly associated with behaviour and determinants. Since policy changed after changes in infections and hospital admissions, this confirms the earlier found influence of increases in hospital admissions on people’s attitudes and behaviour.

**
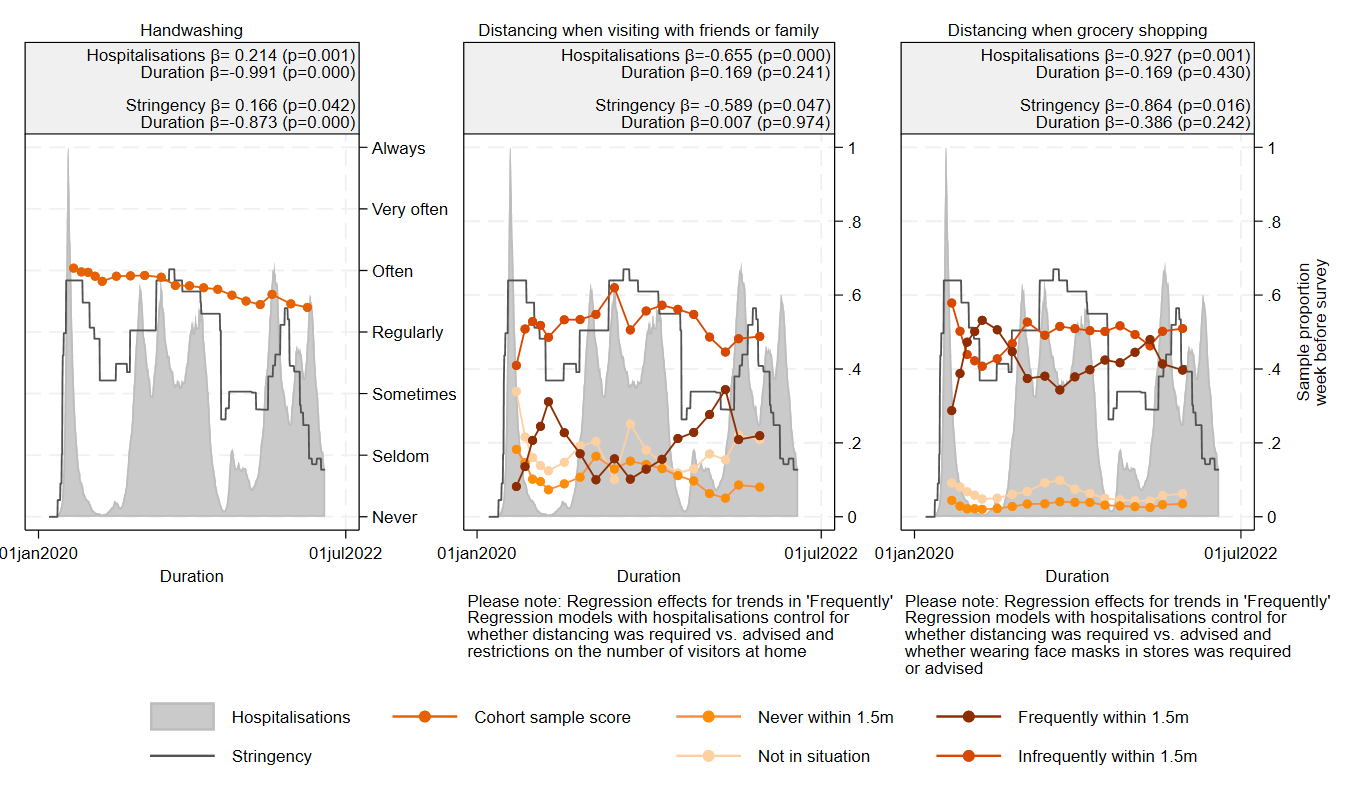
**

**Appendix S4.1: Behaviour (cohort average) with severity (measured with hospitalisations and severity) and duration of the pandemic**


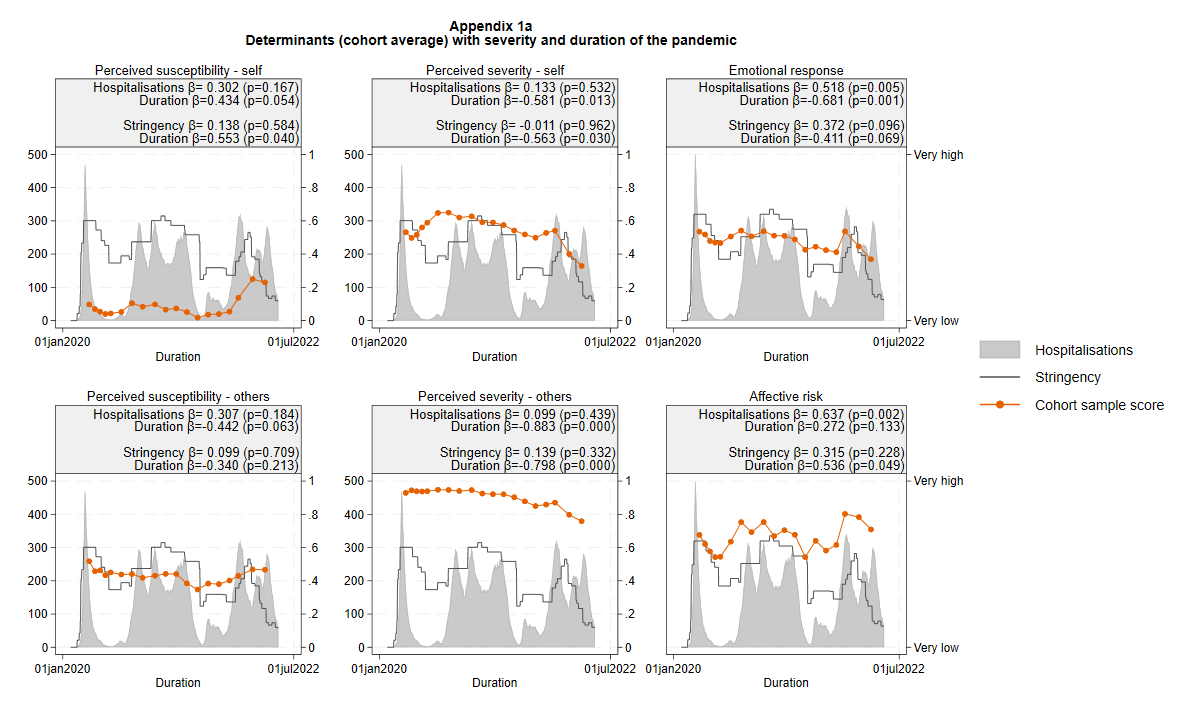


**Appendix S4.2: Determinants (cohort average) with severity (measured with hospitalisations and severity) and duration of the pandemic**


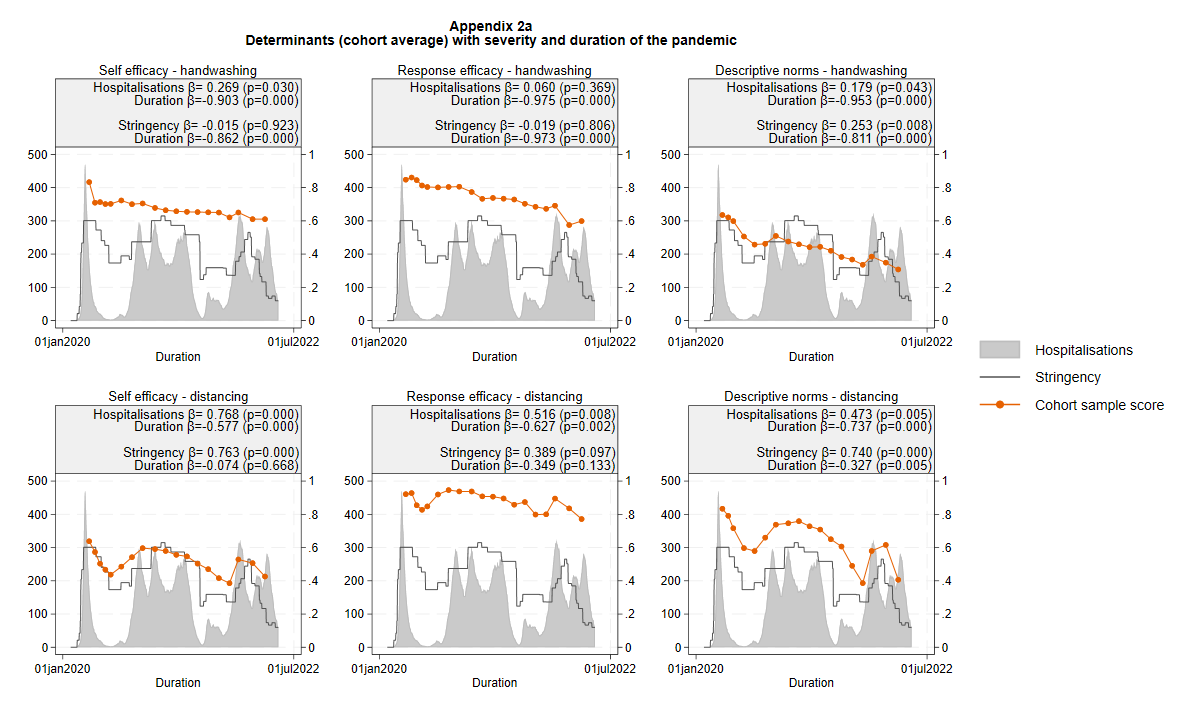


**Appendix S4.2: Determinants (cohort average) with severity (measured with hospitalisations and severity) and duration of the pandemic (continued)**

**Appendix S4.3: Full regression models of behaviour and determinants (cohort averages) with severity (various operationalisations) and duration of the pandemic**

Appendix S4.3.1: Handwashing

|  | Handwashing | Handwashing | Handwashing | Handwashing | Handwashing |
| --- | --- | --- | --- | --- | --- |
| Duration | -0.991^***^ | -1.081^***^ | -1.082^***^ | -0.873^***^ | -0.810^***^ |
| Hospital admissions lagged (7 days) | 0.214^**^ |  |  |  |  |
| Hospital admissions forwarded (7 days) |  | 0.247^**^ |  |  |  |
| Hospital admissions forwarded (14 days) |  |  | 0.222^**^ |  |  |
| Stringency lagged (7 days) |  |  |  | 0.166^*^ |  |
| Stringency forwarded (7 days) |  |  |  |  | 0.242^**^ |
| Observations | 19 | 19 | 19 | 19 | 19 |

Standardized beta coefficients

^*^ *p* < 0.05, ^**^ *p* < 0.01, ^***^ *p* < 0.001

Appendix S4.3.2: Distancing when visiting with friends or family

|  | Not in situation | | | Never within 1.5m | | | Infrequently within 1.5m | | | Frequently within 1.5m | | |
| --- | --- | --- | --- | --- | --- | --- | --- | --- | --- | --- | --- | --- |
| Duration | -0.288 | -0.305 | -0.338 | -0.363^*^ | -0.470^*^ | -0.445 | 0.327 | 0.185 | 0.204 | 0.169 | 0.320 | 0.321 |
| Duration squared |  |  |  |  |  |  |  |  |  |  |  |  |
| Hospital admissions lagged (7 days) | 0.494 |  |  | 0.666^***^ |  |  | 0.002 |  |  | -0.655^***^ |  |  |
| Hospital admissions forwarded (7 days) |  | 0.293 |  |  | 0.577^*^ |  |  | 0.310 |  |  | -0.666^**^ |  |
| Hospital admissions forwarded (14 days) |  |  | 0.319 |  |  | 0.459 |  |  | 0.237 |  |  | -0.586^*^ |
| Policy: distance | 0.191 | 0.196 | 0.169 | -0.188 | -0.257 | -0.231 | -0.392 | -0.521 | -0.503 | 0.184 | 0.293 | 0.290 |
| Policy: visitors | 0.061 | 0.159 | 0.137 | 0.223 | 0.264 | 0.310 | 0.304 | 0.151 | 0.181 | -0.352^*^ | -0.345 | -0.367 |
| Observations | 18 | 18 | 18 | 18 | 18 | 18 | 18 | 18 | 18 | 18 | 18 | 18 |

Standardized beta coefficients

^*^ *p* < 0.05, ^**^ *p* < 0.01, ^***^ *p* < 0.001

Appendix S4.3.3: Distancing when grocery shopping

|  | Not in situation | | | Never within 1.5m | | | Infrequently within 1.5m | | | Frequently within 1.5m | | |
| --- | --- | --- | --- | --- | --- | --- | --- | --- | --- | --- | --- | --- |
| Duration | -0.539^*^ | -0.660^*^ | -0.735^**^ | 0.245 | 0.014 | -0.068 | 0.484^*^ | 0.211 | 0.139 | -0.169 | 0.082 | 0.165 |
| Duration squared |  |  |  |  |  |  |  |  |  |  |  |  |
| Hospital admissions lagged (7 days) | 0.522^*^ |  |  | 0.862^***^ |  |  | 0.970^***^ |  |  | -0.927^***^ |  |  |
| Hospital admissions forwarded (7 days) |  | 0.568 |  |  | 0.641 |  |  | 0.587 |  |  | -0.664 |  |
| Hospital admissions forwarded (14 days) |  |  | 0.488 |  |  | 0.452 |  |  | 0.329 |  |  | -0.442 |
| Policy: distance | -0.094 | -0.189 | -0.161 | -0.301 | -0.346 | -0.276 | -0.355 | -0.355 | -0.259 | 0.310 | 0.349 | 0.267 |
| Policy: face masks | 0.268 | 0.252 | 0.380 | -0.012 | 0.186 | 0.391 | -0.407 | -0.082 | 0.158 | 0.175 | -0.057 | -0.286 |
| Observations | 18 | 18 | 18 | 18 | 18 | 18 | 18 | 18 | 18 | 18 | 18 | 18 |

Standardized beta coefficients

^*^ *p* < 0.05, ^**^ *p* < 0.01, ^***^ *p* < 0.001

Appendix S4.3.4: Distancing when visiting with friends or family

|  | Not in situation |  | Never within 1.5m |  | Infrequently within 1.5m |  | Frequently within 1.5m |  |
| --- | --- | --- | --- | --- | --- | --- | --- | --- |
| Duration | -0.164 | -0.081 | -0.196 | -0.053 | 0.326 | 0.352 | 0.007 | -0.134 |
| Stringency lagged (7 days) | 0.496 |  | 0.682^*^ |  | -0.132 |  | -0.589^*^ |  |
| Stringency forwarded (7 days) |  | 0.528 |  | 0.900^**^ |  | 0.146 |  | -0.879^**^ |
| Policy: distance | 0.334 | 0.288 | 0.005 | -0.068 | -0.396 | -0.400 | -0.003 | 0.066 |
| Policy: visitors | -0.065 | -0.081 | 0.042 | -0.106 | 0.404 | 0.199 | -0.236 | -0.034 |
| Observations | 18 | 18 | 18 | 18 | 18 | 18 | 18 | 18 |

Standardized beta coefficients

^*^ *p* < 0.05, ^**^ *p* < 0.01, ^***^ *p* < 0.001

Appendix S4.3.5: Distancing when grocery shopping

|  | Not in situation |  | Never within 1.5m |  | Infrequently within 1.5m |  | Frequently within 1.5m |  |
| --- | --- | --- | --- | --- | --- | --- | --- | --- |
| Duration | -0.167 | 0.130 | 0.352 | 0.771^*^ | 0.594 | 0.959^*^ | -0.386 | -0.779^*^ |
| Stringency lagged (7 days) | 0.889^***^ |  | 0.651^*^ |  | 0.716 |  | -0.864^*^ |  |
| Stringency forwarded (7 days) |  | 1.074^***^ |  | 1.043^**^ |  | 1.024^*^ |  | -1.177^**^ |
| Policy: distance | 0.195 | 0.118 | 0.020 | -0.013 | 0.003 | -0.045 | -0.066 | -0.004 |
| Policy: face masks | 0.030 | -0.137 | 0.194 | -0.124 | -0.163 | -0.419 | 0.074 | 0.338 |
| Observations | 18 | 18 | 18 | 18 | 18 | 18 | 18 | 18 |

Standardized beta coefficients

^*^ *p* < 0.05, ^**^ *p* < 0.01, ^***^ *p* < 0.001

Appendix S4.3.6: Emotional response

|  | Emotional response | Emotional response | Emotional response | Emotional response | Emotional response |
| --- | --- | --- | --- | --- | --- |
| Duration | -0.681^***^ | -0.943^***^ | -0.997^***^ | -0.411 | -0.194 |
| Hospital admissions lagged (7 days) | 0.518^**^ |  |  |  |  |
| Hospital admissions forwarded (7 days) |  | 0.684^***^ |  |  |  |
| Hospital admissions forwarded (14 days) |  |  | 0.705^***^ |  |  |
| Stringency lagged (7 days) |  |  |  | 0.372 |  |
| Stringency forwarded (7 days) |  |  |  |  | 0.668^**^ |
| Observations | 19 | 19 | 19 | 19 | 19 |

Standardized beta coefficients

^*^ *p* < 0.05, ^**^ *p* < 0.01, ^***^ *p* < 0.001

Appendix S4.3.7: Affective risk

|  | Affective risk | Affective risk | Affective risk | Affective risk | Affective risk |
| --- | --- | --- | --- | --- | --- |
| Duration | 0.272 | -0.074 | -0.182 | 0.536^*^ | 0.693^*^ |
| Hospital admissions lagged (7 days) | 0.637^**^ |  |  |  |  |
| Hospital admissions forwarded (7 days) |  | 0.888^***^ |  |  |  |
| Hospital admissions forwarded (14 days) |  |  | 0.980^***^ |  |  |
| Stringency lagged (7 days) |  |  |  | 0.315 |  |
| Stringency forwarded (7 days) |  |  |  |  | 0.520 |
| Observations | 19 | 19 | 19 | 19 | 19 |

Standardized beta coefficients

^*^ *p* < 0.05, ^**^ *p* < 0.01, ^***^ *p* < 0.001

Appendix S4.3.8: Perceived susceptibility - self

|  | Perceived susceptibility - self | Perceived susceptibility - self | Perceived susceptibility - self | Perceived susceptibility - self | Perceived susceptibility - self |
| --- | --- | --- | --- | --- | --- |
| Duration | 0.434 | 0.246 | 0.156 | 0.553^*^ | 0.484 |
| Hospital admissions lagged (7 days) | 0.302 |  |  |  |  |
| Hospital admissions forwarded (7 days) |  | 0.466 |  |  |  |
| Hospital admissions forwarded (14 days) |  |  | 0.573^*^ |  |  |
| Stringency lagged (7 days) |  |  |  | 0.138 |  |
| Stringency forwarded (7 days) |  |  |  |  | -0.005 |
| Observations | 19 | 19 | 19 | 19 | 19 |

Standardized beta coefficients

^*^ *p* < 0.05, ^**^ *p* < 0.01, ^***^ *p* < 0.001

Appendix S4.3.9: Perceived susceptibility - others

|  | Perceived susceptibility - others | Perceived susceptibility - others | Perceived susceptibility - others | Perceived susceptibility - others | Perceived susceptibility - others |
| --- | --- | --- | --- | --- | --- |
| Duration | -0.442 | -0.619^*^ | -0.700^*^ | -0.340 | -0.420 |
| Hospital admissions lagged (7 days) | 0.307 |  |  |  |  |
| Hospital admissions forwarded (7 days) |  | 0.447 |  |  |  |
| Hospital admissions forwarded (14 days) |  |  | 0.542^*^ |  |  |
| Stringency lagged (7 days) |  |  |  | 0.099 |  |
| Stringency forwarded (7 days) |  |  |  |  | -0.055 |
| Observations | 19 | 19 | 19 | 19 | 19 |

Standardized beta coefficients

^*^ *p* < 0.05, ^**^ *p* < 0.01, ^***^ *p* < 0.001

Appendix S4.3.10: Perceived severity - self

|  | Perceived severity - self | Perceived severity - self | Perceived severity - self | Perceived severity - self | Perceived severity - self |
| --- | --- | --- | --- | --- | --- |
| Duration | -0.581^*^ | -0.683^*^ | -0.720^**^ | -0.563^*^ | -0.344 |
| Hospital admissions lagged (7 days) | 0.133 |  |  |  |  |
| Hospital admissions forwarded (7 days) |  | 0.244 |  |  |  |
| Hospital admissions forwarded (14 days) |  |  | 0.282 |  |  |
| Stringency lagged (7 days) |  |  |  | -0.011 |  |
| Stringency forwarded (7 days) |  |  |  |  | 0.360 |
| Observations | 19 | 19 | 19 | 19 | 19 |

Standardized beta coefficients

^*^ *p* < 0.05, ^**^ *p* < 0.01, ^***^ *p* < 0.001

Appendix S4.3.11: Perceived severity - others

|  | Perceived severity - others | Perceived severity - others | Perceived severity - others | Perceived severity - others | Perceived severity - others |
| --- | --- | --- | --- | --- | --- |
| Duration | -0.883^***^ | -0.941^***^ | -0.943^***^ | -0.798^***^ | -0.665^***^ |
| Hospital admissions lagged (7 days) | 0.099 |  |  |  |  |
| Hospital admissions forwarded (7 days) |  | 0.147 |  |  |  |
| Hospital admissions forwarded (14 days) |  |  | 0.135 |  |  |
| Stringency lagged (7 days) |  |  |  | 0.139 |  |
| Stringency forwarded (7 days) |  |  |  |  | 0.337^*^ |
| Observations | 19 | 19 | 19 | 19 | 19 |

Standardized beta coefficients

^*^ *p* < 0.05, ^**^ *p* < 0.01, ^***^ *p* < 0.001

Appendix S4.3.12: Self efficacy - handwashing

|  | Self efficacy - handwashing | Self efficacy - handwashing | Self efficacy - handwashing | Self efficacy - handwashing | Self efficacy - handwashing |
| --- | --- | --- | --- | --- | --- |
| Duration | -0.903^***^ | -0.919^***^ | -0.893^***^ | -0.862^***^ | -0.833^***^ |
| Hospital admissions lagged (7 days) | 0.269^*^ |  |  |  |  |
| Hospital admissions forwarded (7 days) |  | 0.124 |  |  |  |
| Hospital admissions forwarded (14 days) |  |  | 0.065 |  |  |
| Stringency lagged (7 days) |  |  |  | -0.015 |  |
| Stringency forwarded (7 days) |  |  |  |  | 0.038 |
| Observations | 19 | 19 | 19 | 19 | 19 |

Standardized beta coefficients

^*^ *p* < 0.05, ^**^ *p* < 0.01, ^***^ *p* < 0.001

Appendix S4.3.13: Self efficacy - distancing

|  | Self efficacy - distancing | Self efficacy - distancing | Self efficacy - distancing | Self efficacy - distancing | Self efficacy - distancing |
| --- | --- | --- | --- | --- | --- |
| Duration | -0.577^***^ | -0.830^***^ | -0.842^**^ | -0.074 | 0.107 |
| Hospital admissions lagged (7 days) | 0.768^***^ |  |  |  |  |
| Hospital admissions forwarded (7 days) |  | 0.752^***^ |  |  |  |
| Hospital admissions forwarded (14 days) |  |  | 0.694^**^ |  |  |
| Stringency lagged (7 days) |  |  |  | 0.763^***^ |  |
| Stringency forwarded (7 days) |  |  |  |  | 0.925^***^ |
| Observations | 19 | 19 | 19 | 19 | 19 |

Standardized beta coefficients

^*^ *p* < 0.05, ^**^ *p* < 0.01, ^***^ *p* < 0.001

Appendix S4.3.14: Response efficacy - handwashing

|  | Response efficacy - handwashing | Response efficacy - handwashing | Response efficacy - handwashing | Response efficacy - handwashing | Response efficacy - handwashing |
| --- | --- | --- | --- | --- | --- |
| Duration | -0.975^***^ | -1.000^***^ | -0.984^***^ | -0.973^***^ | -0.933^***^ |
| Hospital admissions lagged (7 days) | 0.060 |  |  |  |  |
| Hospital admissions forwarded (7 days) |  | 0.070 |  |  |  |
| Hospital admissions forwarded (14 days) |  |  | 0.034 |  |  |
| Stringency lagged (7 days) |  |  |  | -0.019 |  |
| Stringency forwarded (7 days) |  |  |  |  | 0.052 |
| Observations | 19 | 19 | 19 | 19 | 19 |

Standardized beta coefficients

^*^ *p* < 0.05, ^**^ *p* < 0.01, ^***^ *p* < 0.001

Appendix S4.3.15: Response efficacy - distancing

|  | Response efficacy - distancing | Response efficacy - distancing | Response efficacy - distancing | Response efficacy - distancing | Response efficacy - distancing |
| --- | --- | --- | --- | --- | --- |
| Duration | -0.627^**^ | -0.856^***^ | -0.897^***^ | -0.349 | -0.128 |
| Hospital admissions lagged (7 days) | 0.516^**^ |  |  |  |  |
| Hospital admissions forwarded (7 days) |  | 0.619^**^ |  |  |  |
| Hospital admissions forwarded (14 days) |  |  | 0.624^**^ |  |  |
| Stringency lagged (7 days) |  |  |  | 0.389 |  |
| Stringency forwarded (7 days) |  |  |  |  | 0.688^**^ |
| Observations | 19 | 19 | 19 | 19 | 19 |

Standardized beta coefficients

^*^ *p* < 0.05, ^**^ *p* < 0.01, ^***^ *p* < 0.001

Appendix S4.3.16: Descriptive norms - handwashing

|  | Descriptive norms - handwashing | Descriptive norms - handwashing | Descriptive norms - handwashing | Descriptive norms - handwashing | Descriptive norms - handwashing |
| --- | --- | --- | --- | --- | --- |
| Duration | -0.953^***^ | -0.985^***^ | -0.961^***^ | -0.811^***^ | -0.817^***^ |
| Hospital admissions lagged (7 days) | 0.179^*^ |  |  |  |  |
| Hospital admissions forwarded (7 days) |  | 0.107 |  |  |  |
| Hospital admissions forwarded (14 days) |  |  | 0.051 |  |  |
| Stringency lagged (7 days) |  |  |  | 0.253^**^ |  |
| Stringency forwarded (7 days) |  |  |  |  | 0.191 |
| Observations | 18 | 18 | 18 | 18 | 18 |

Standardized beta coefficients

^*^ *p* < 0.05, ^**^ *p* < 0.01, ^***^ *p* < 0.001

Appendix S4.3.17: Descriptive norms - distancing

|  | Descriptive norms - distancing | Descriptive norms - distancing | Descriptive norms - distancing | Descriptive norms - distancing | Descriptive norms - distancing |
| --- | --- | --- | --- | --- | --- |
| Duration | -0.737^***^ | -0.876^***^ | -0.863^***^ | -0.327^**^ | -0.192 |
| Hospital admissions lagged (7 days) | 0.473^**^ |  |  |  |  |
| Hospital admissions forwarded (7 days) |  | 0.399 |  |  |  |
| Hospital admissions forwarded (14 days) |  |  | 0.327 |  |  |
| Stringency lagged (7 days) |  |  |  | 0.740^***^ |  |
| Stringency forwarded (7 days) |  |  |  |  | 0.812^***^ |
| Observations | 18 | 18 | 18 | 18 | 18 |

Standardized beta coefficients

^*^ *p* < 0.05, ^**^ *p* < 0.01, ^***^ *p* < 0.001

**Appendix S5: FE-associations between psychosocial determinants and distancing (marginal effects, relative to ‘frequently <1.5m from others)**

**
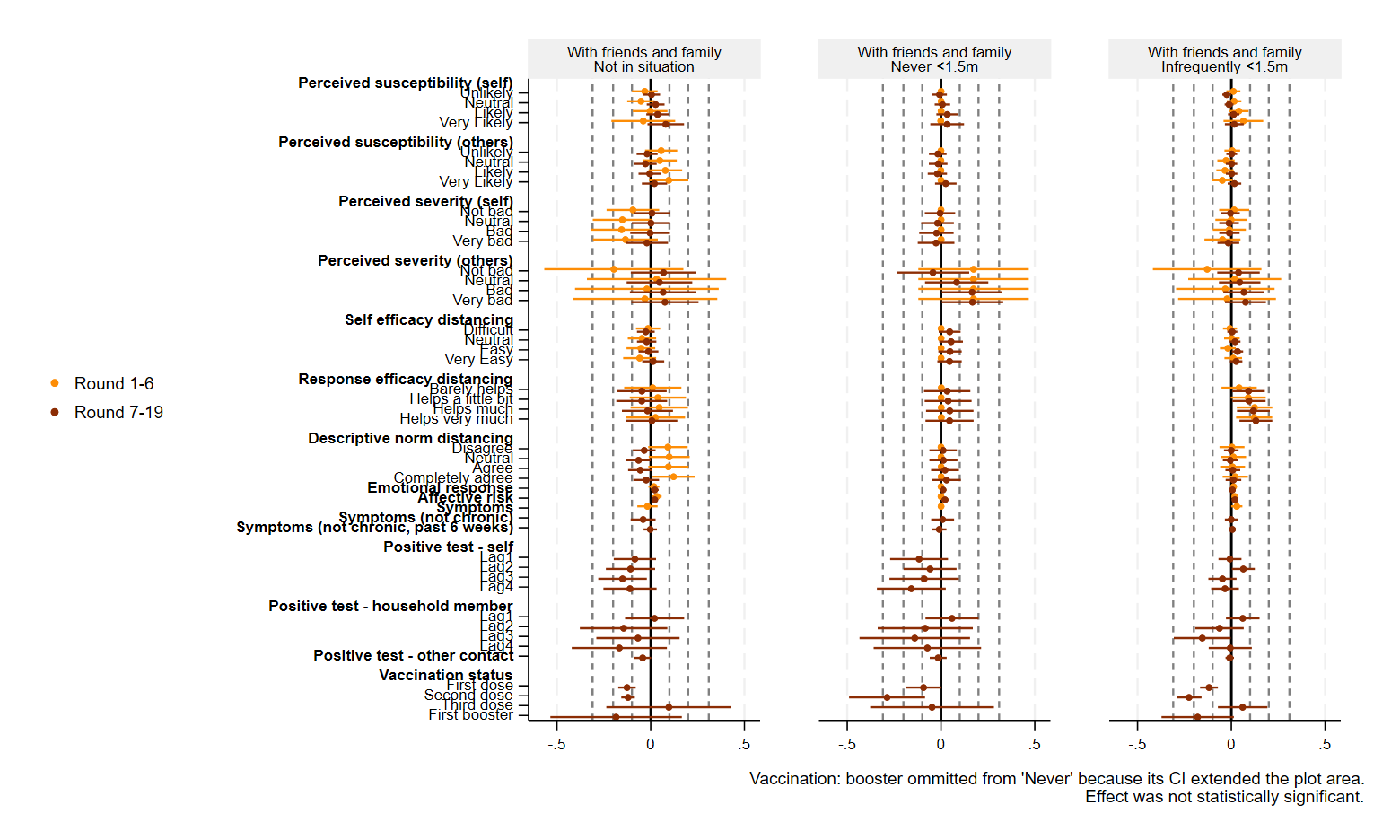
**

**
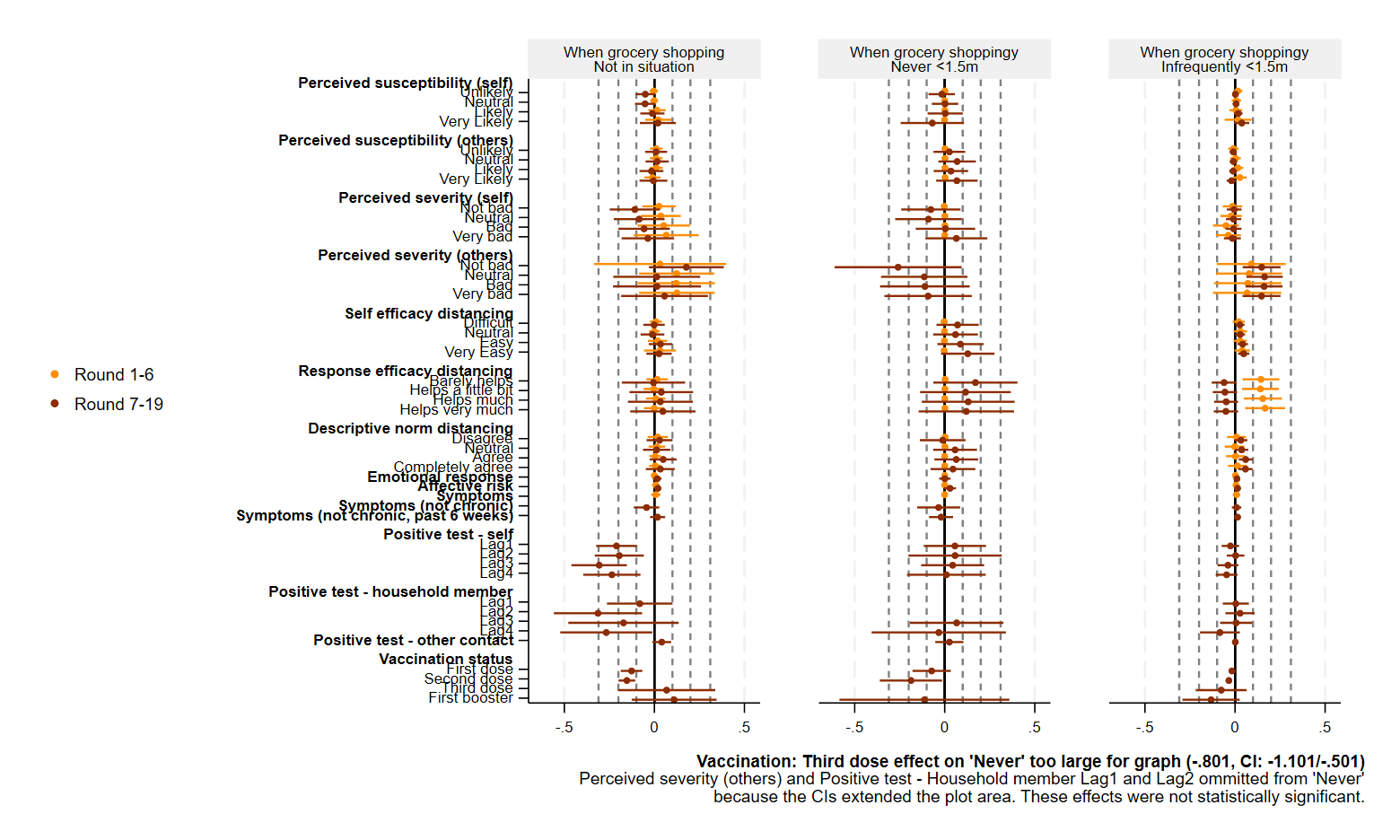
**

**Appendix S6: FE-associations between psychosocial determinants and behaviour moderated by severity and duration of the pandemic
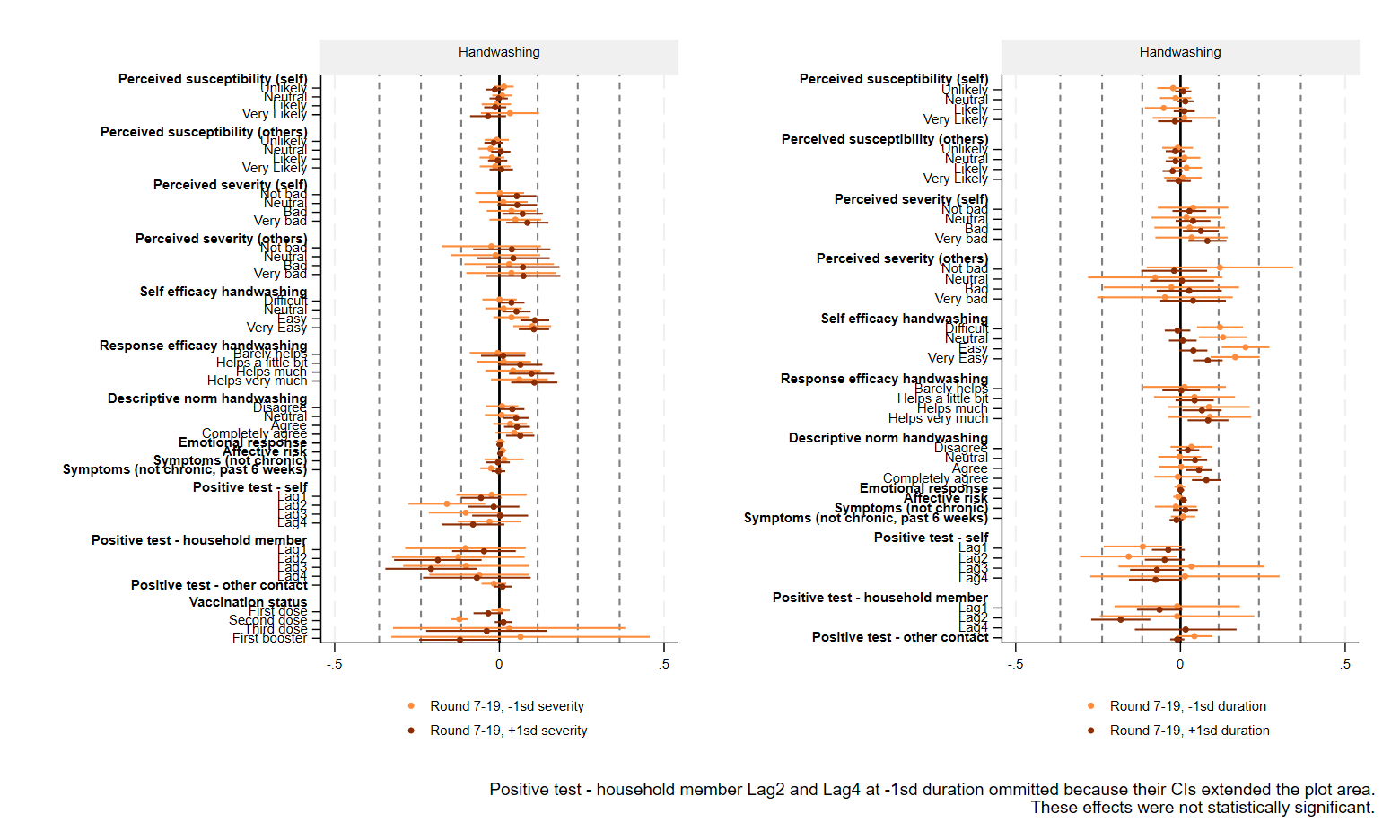
**

**
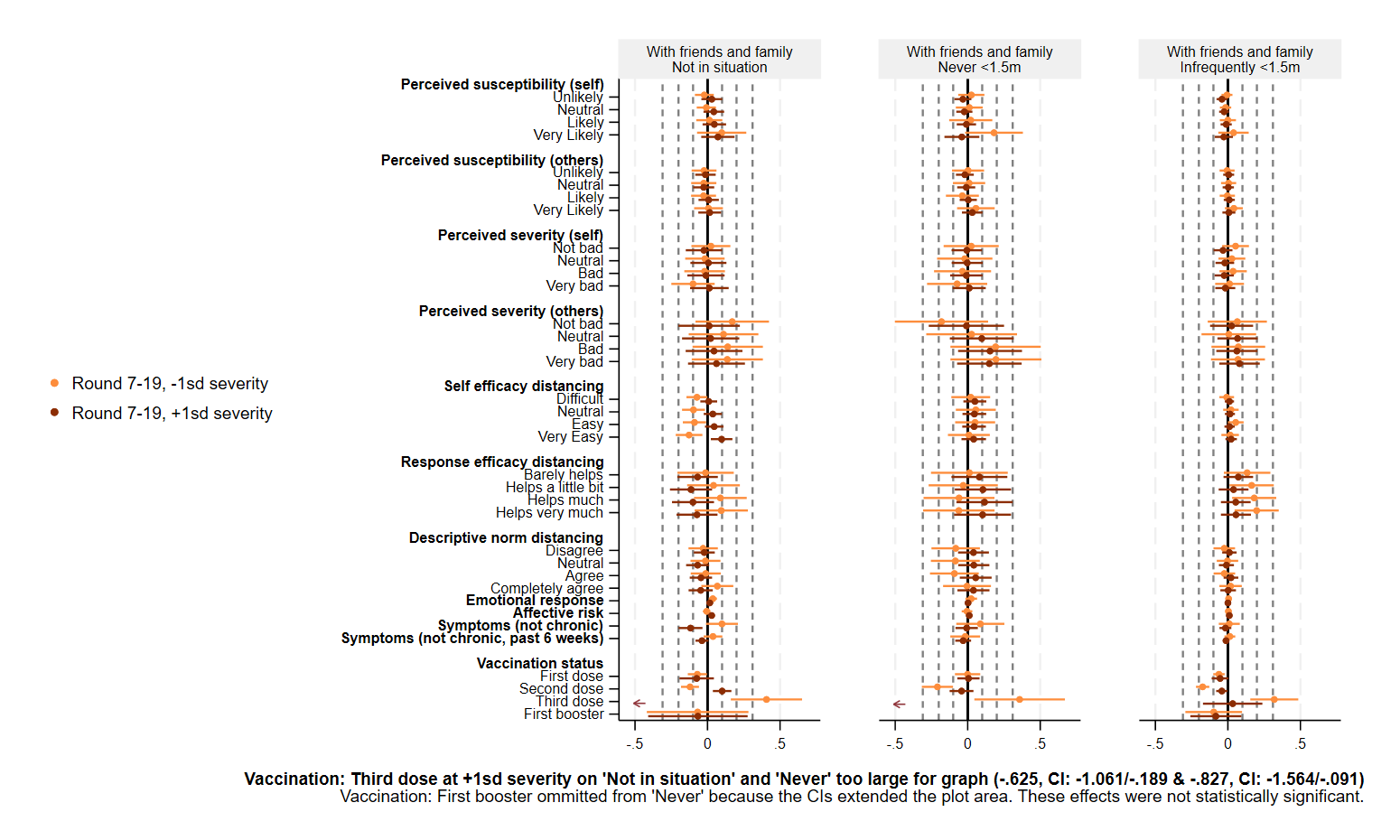
**

**
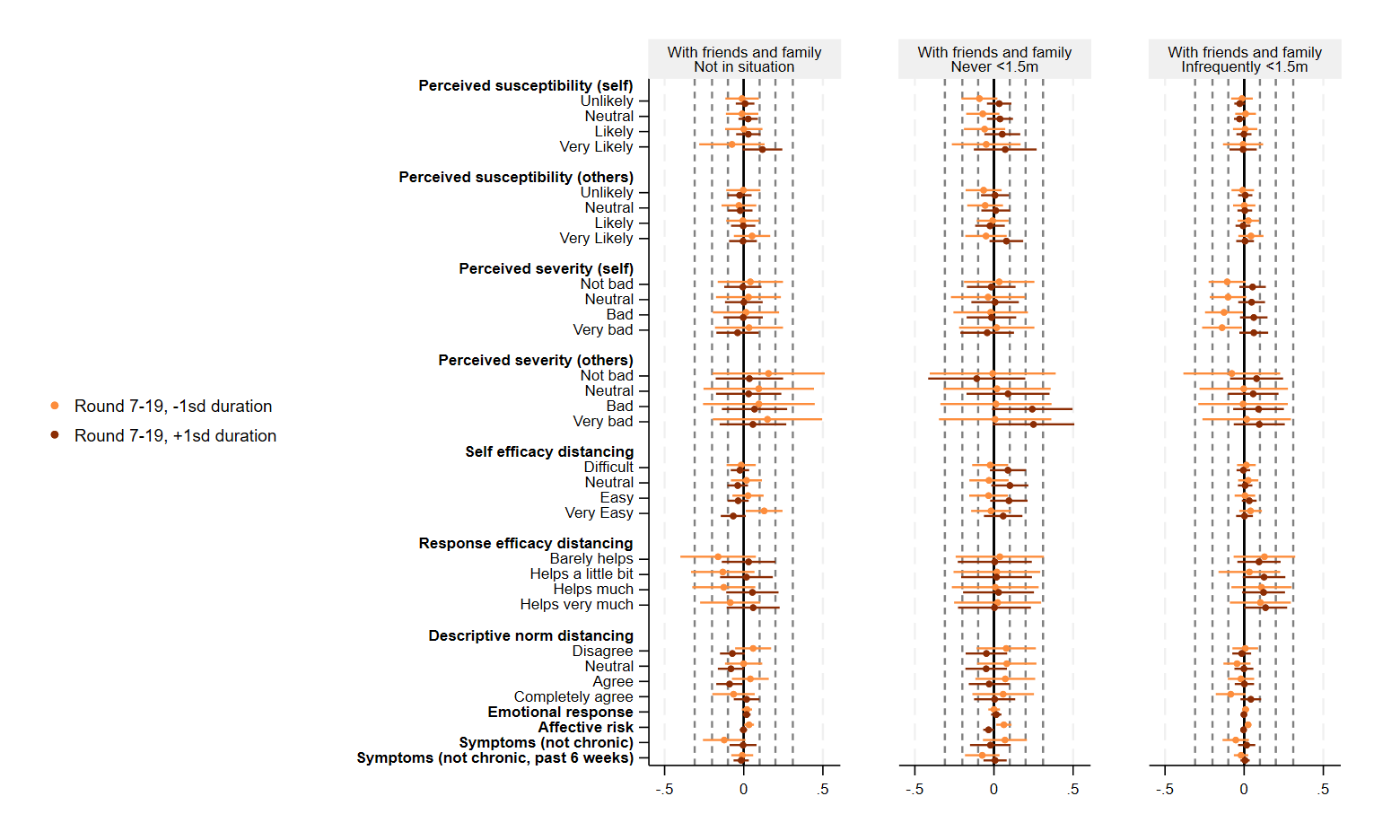
**

**
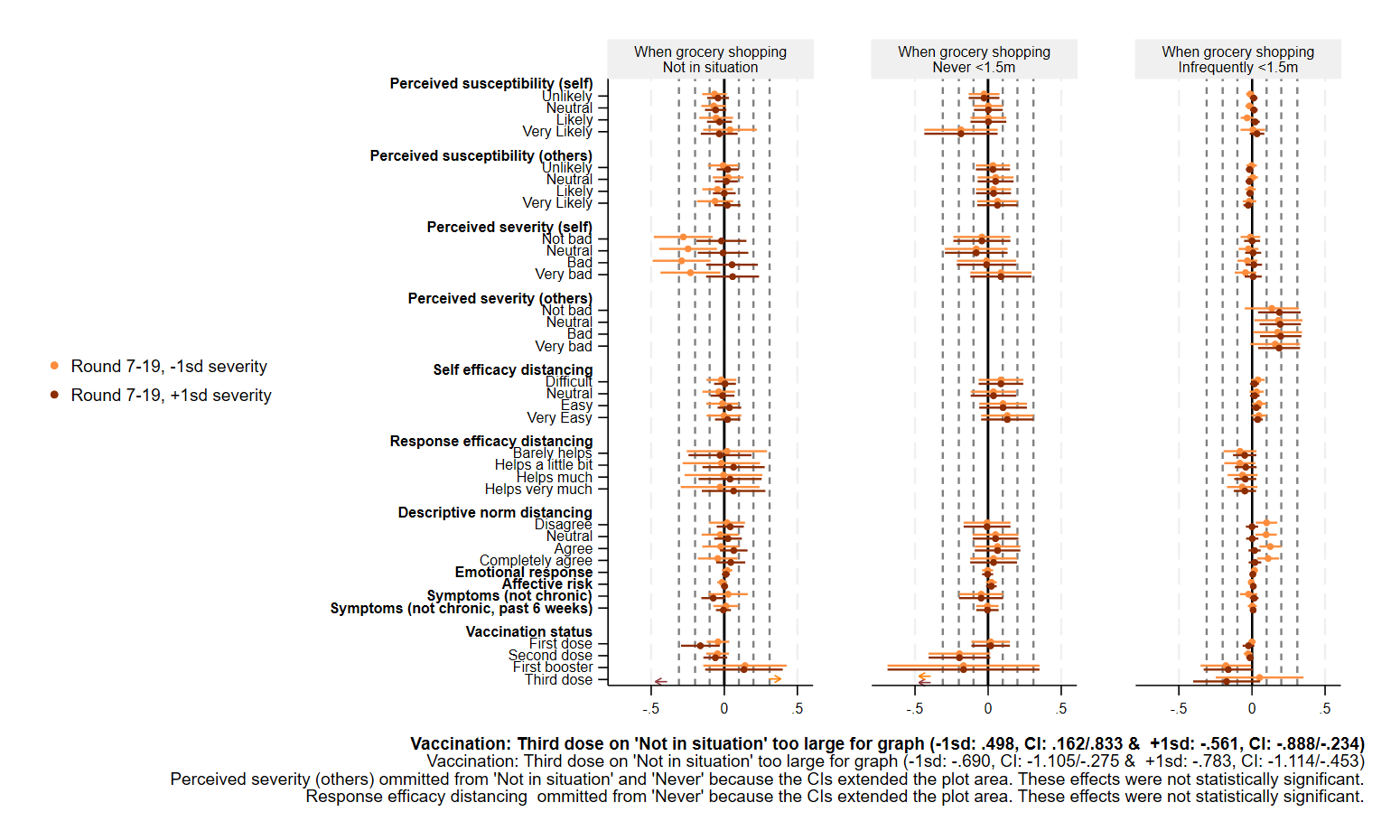
**

**
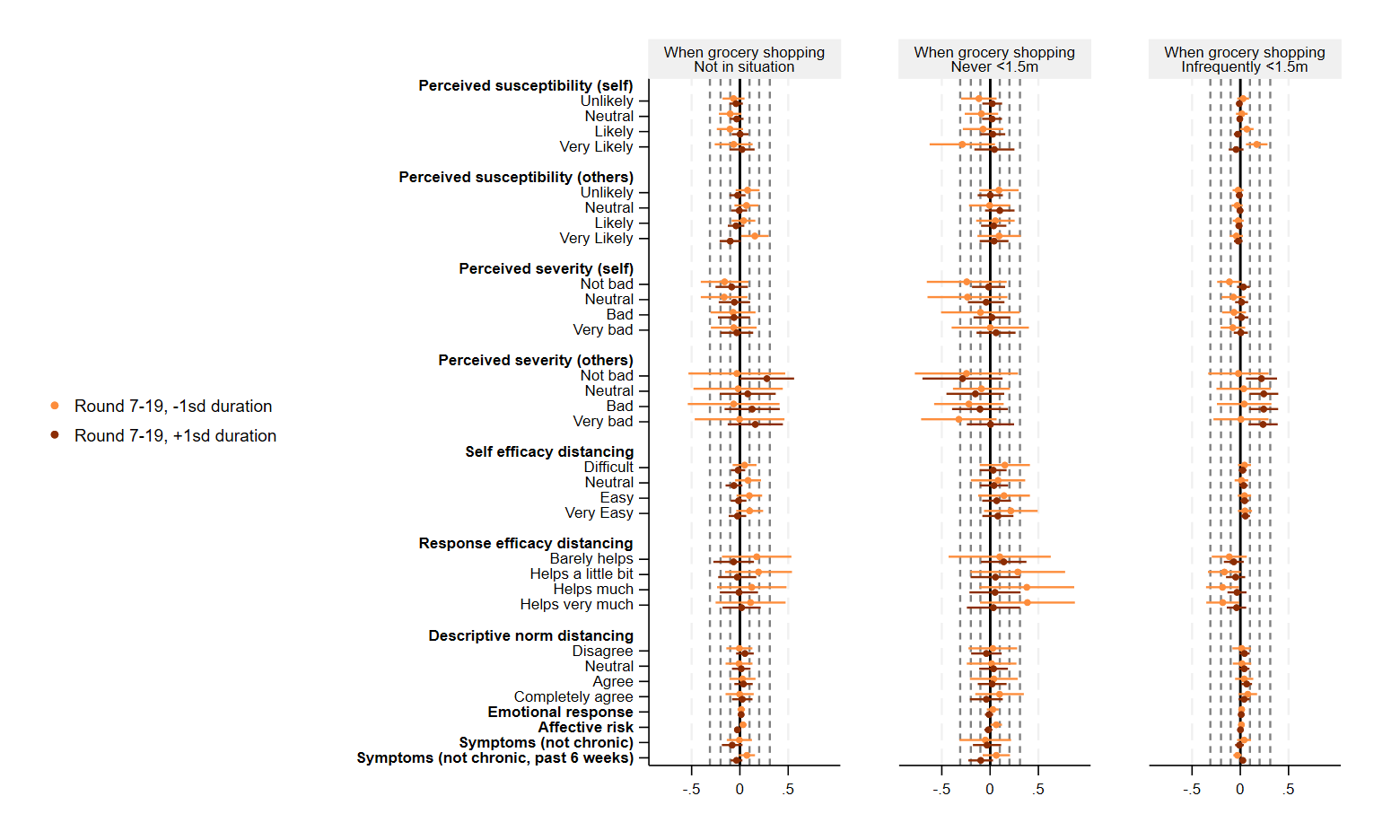
**

**Appendix S7: FE-associations between psychosocial determinants and behaviour – measured concurrently**


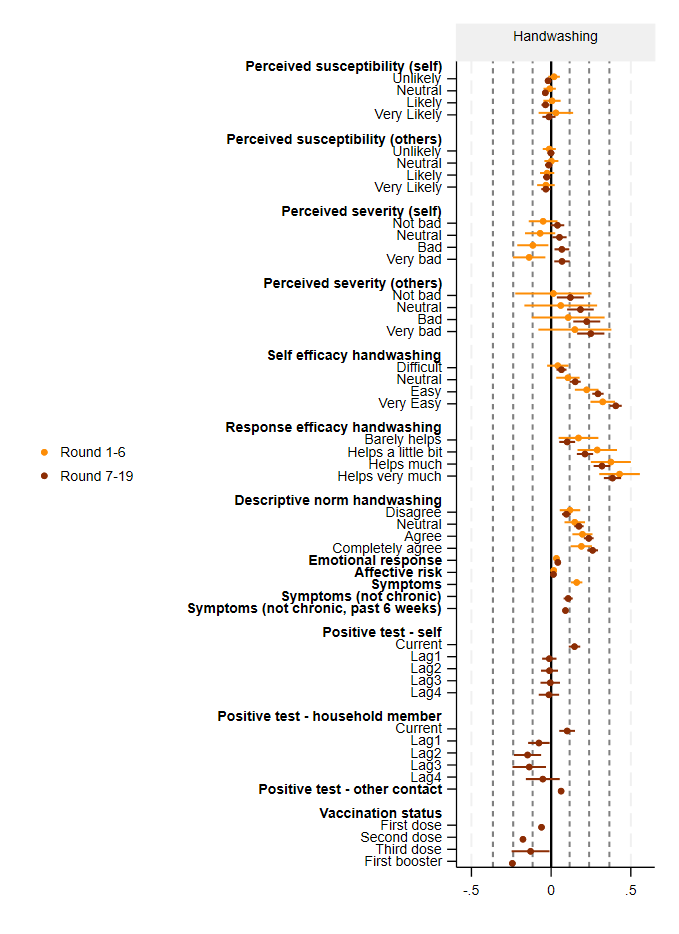

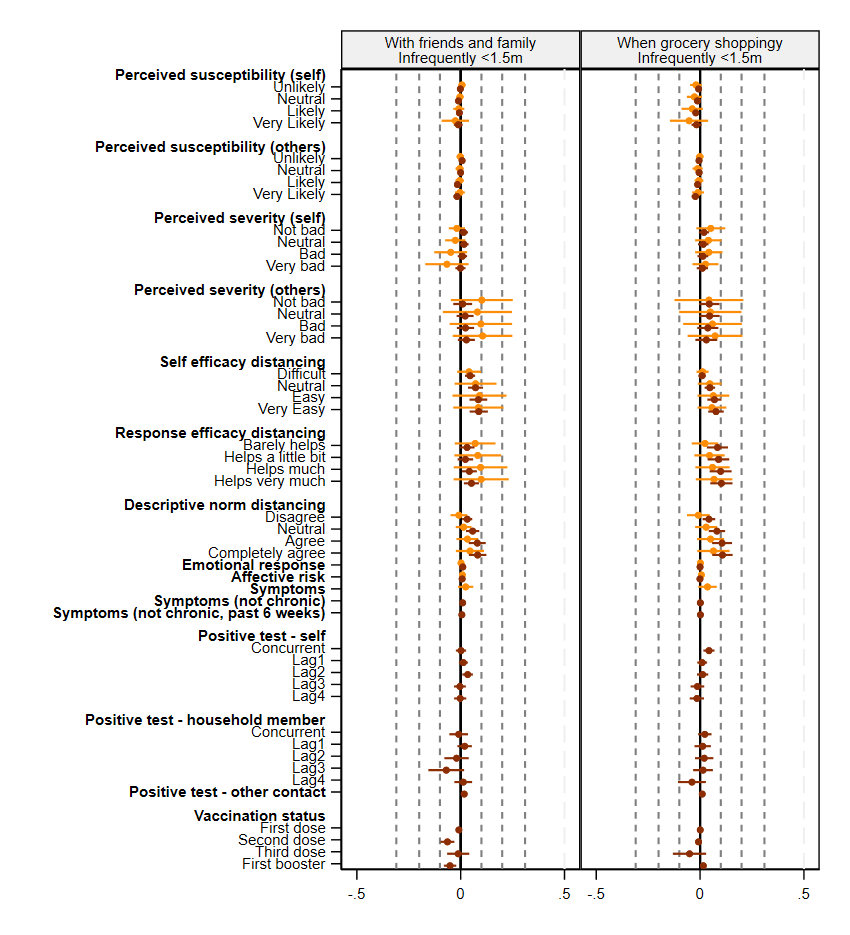


**Appendix S8: Between-within model for associations between psychosocial determinants and handwashing^[[3]](#footnote-3)^**
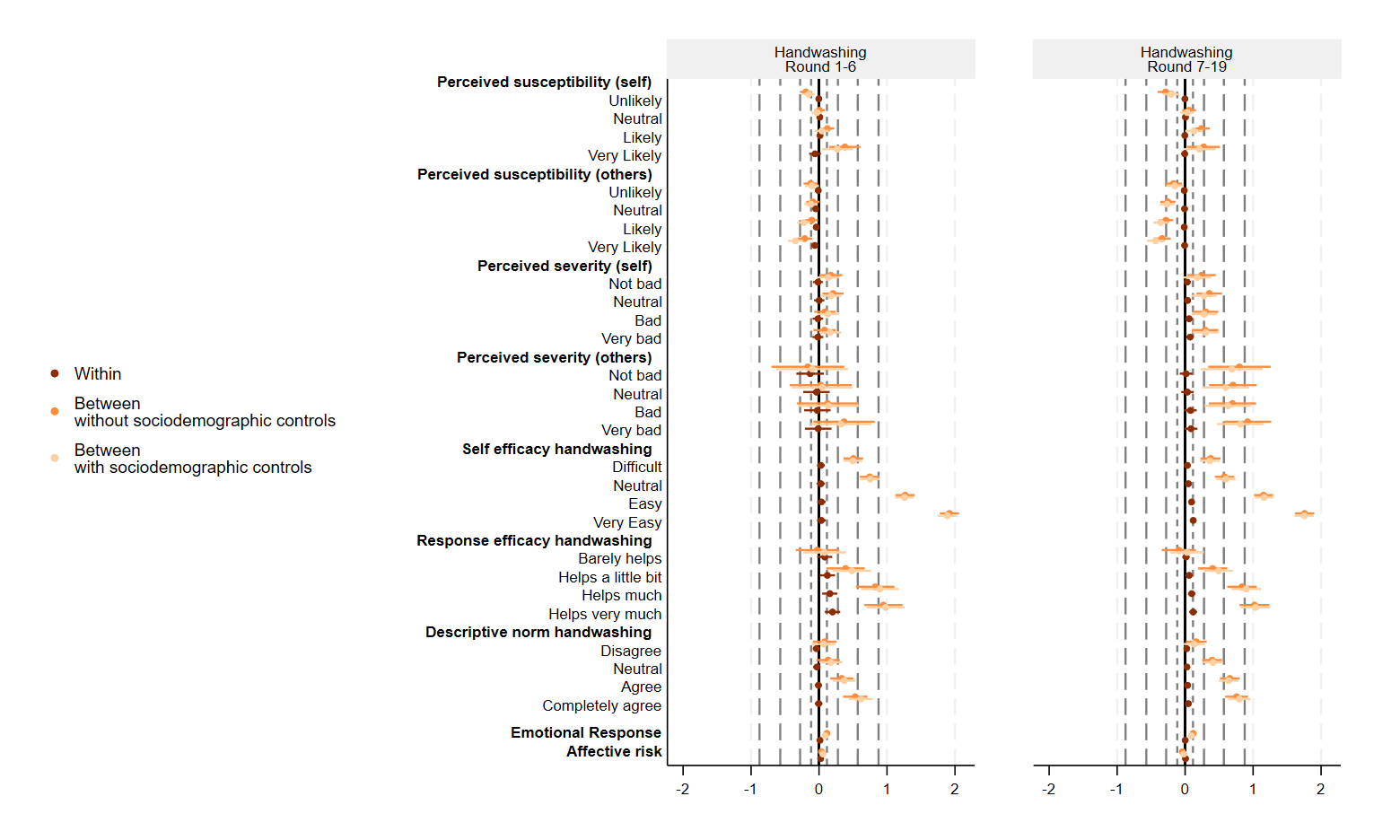


**Appendix S9: FE-associations between psychosocial determinants and behaviour – determinants included separately**

**
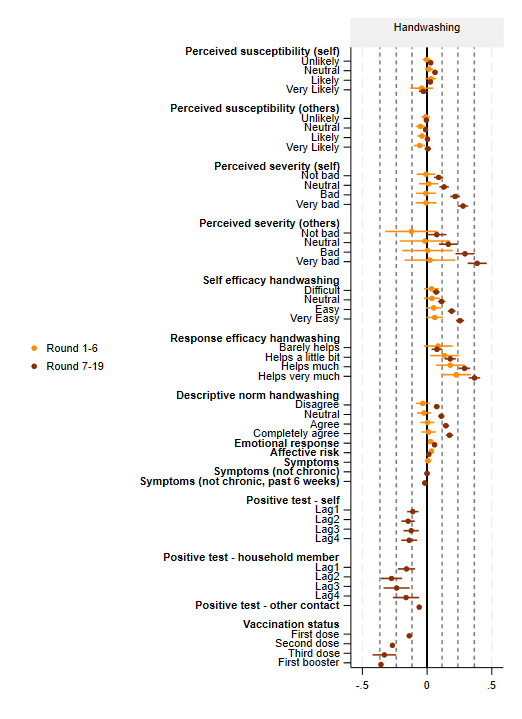

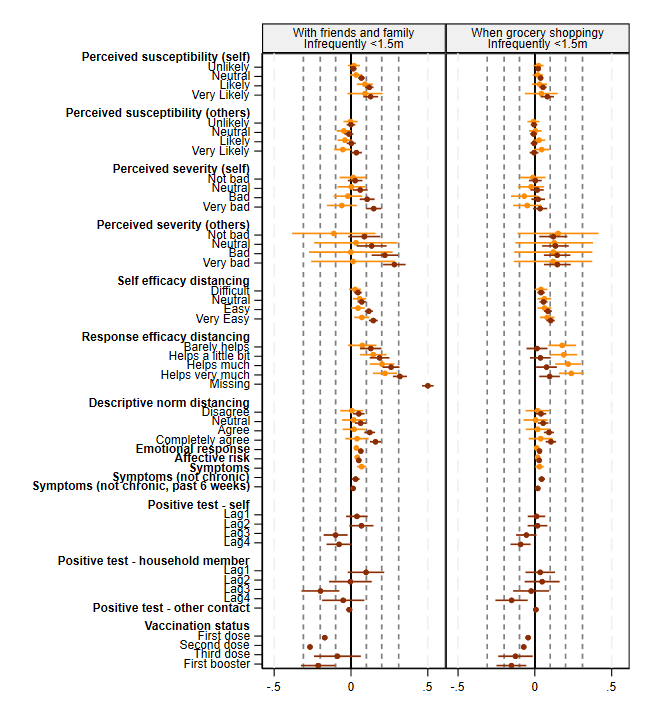
**

1. Bussemakers, C., van Dijk, M., Dima, A. L., & de Bruin, M. (2022). *How well do surveys on adherence to pandemic policies assess actual behaviour: measurement properties of the Dutch Covid-19 Adherence to Prevention Advice Survey (CAPAS)*. https://osf.io/rm8qn/ [↑](#footnote-ref-1)
2. de Vet, H. C. W., Terwee, C. B., Mokkink, L. B., & Knol, D. L. (2011). *Measurement in Medicine: A Practical Guide*. Cambridge University Press. https://doi.org/DOI: 10.1017/CBO9780511996214 [↑](#footnote-ref-2)
3. Models with and without sociodemographic factors illustrate the relevance of unobserved confounding factors: even with controls, between-effects differ substantially from the within-effects. Moreover, although cues to action were included in the model to ensure sufficient control, these effects are not presented as they are inherently time-specific so there is no meaningful interpretation of between-effects. [↑](#footnote-ref-3)
